# Variation in the Chemical Composition and Biological Activities of Propolis Collected During Two Seasonal Periods in the Zona da Mata Region of Brazil

**DOI:** 10.3390/molecules31203611

**Published:** 2026-10-10

**Authors:** Laís A. Moreira, João Paulo B. Sousa, Carla Monteiro Leal, Victor Campana Leite, Jessica Pereira Silva, Maria Luiza A. A. Soares, Gislaine Gonçalves Rocha, Paula Marynella Alves Pereira Lima, Fabíola Dutra Rocha, Wesley W. G. Nascimento, Nícolas de Castro Campos Pinto, Humberto Moreira Húngaro, Thaise Gonçalves Araújo, Ademar A. Da Silva Filho

**Affiliations:** 1 Departamento de Ciências Farmacêuticas, Faculdade de Farmácia, Universidade Federal de Juiz de Fora, R. José Lourenço Kelmer s/n, Campus Universitário, Juiz de Fora 36036-900, MG, Brazil; lais.moreira@estudante.ufjf.br (L.A.M.); fabiola.rocha@ufjf.br (F.D.R.); humbertomh@gmail.com (H.M.H.); 2 Departamento de Farmácia, Faculdade de Farmácia, Universidade de Brasília, Asa Norte, Campus Darcy Ribeiro, Brasília 70910-900, DF, Brazil; jpsousa595@gmail.com; 3 Departamento de Produtos Naturais e Alimentos, Faculdade de Farmácia, Universidade Federal do Rio de Janeiro, Av. Carlos Chagas Filho, 373, Cidade Universitária, Rio de Janeiro 21941-902, RJ, Brazil; farmaceuticacarlaleal@gmail.com; 4 Laboratório de Produtos Naturais Bioativos, Departamento de Bioquímica, Instituto de Ciências Biológicas, Universidade Federal de Juiz de Fora, R. José Lourenço Kelmer s/n, Campus Universitário, Juiz de Fora 36036-900, MG, Brazil; victor.campana@ufjf.br (V.C.L.); jessicapereira.silva@estudante.ufjf.br (J.P.S.); marialuiza.aasoares@gmail.com (M.L.A.A.S.); nicolas.pinto@ufjf.br (N.d.C.C.P.); 5 Laboratório de Genética e Biotecnologia, Instituto de Biotecnologia, Universidade Federal de Uberlândia, R. Major Jerônimo, 566, Patos de Minas 38700-002, MG, Brazil; gislaineg.rocha08@gmail.com (G.G.R.); paulamapl0607@gmail.com (P.M.A.P.L.); thaisegaraujo@gmail.com (T.G.A.); 6 Departamento de Farmácia, Instituto de Ciências da Vida, Universidade Federal de Juiz de Fora, R. Manoel Byrro, 241, Campus Governador Valadares, Governador Valadares 35032-620, MG, Brazil; wesley.nascimento@ufjf.br

**Keywords:** Brazilian propolis, phenolic compounds, artepillin C, antioxidant activity, antibacterial activity, UHPLC-ESI-MS/MS, cytotoxicity

## Abstract

Propolis is a chemically complex bee product whose composition and biological properties are influenced by botanical and environmental factors. This study compared eight propolis samples collected during summer and winter from four apiaries in the Zona da Mata region of Minas Gerais, Brazil. Samples were characterized by physicochemical properties, phenolic and flavonoid content, qualitative chemical profiles by UHPLC-ESI-MS/MS, and electrical impedance, while antioxidant, antibacterial, and cytotoxic activities were evaluated using complementary in vitro assays. Summer-collected samples generally exhibited higher dry extract and total phenolic content and lower wax levels, whereas flavonoid content remained comparatively stable. Differences were also observed in electrical impedance and several antioxidant responses, with total phenolic content strongly and negatively correlated with DPPH IC_50_ values (*ρ* = −0.864). All extracts inhibited *Staphylococcus aureus*, predominantly with MIC (Minimal Inhibitory Concentration) values of 250–500 µg/mL, without detectable bactericidal activity at the concentrations tested. UHPLC-ESI-MS/MS profiling revealed qualitatively similar chemical profiles comprising phenolic and prenylated derivatives, including artepillin C, consistent with a possible contribution from *Baccharis dracunculifolia*. Notably, the samples from D apiary (D1 and D2) showed pronounced and selective cytotoxicity against MDA-MB-231 cells. Overall, differences in chemical, physicochemical, and biological properties were observed between the two collection periods of regional propolis.

## 1. Introduction

Propolis is a resinous natural product produced by honeybees through the collection of plant exudates, which are subsequently mixed with wax and salivary enzymes inside the hive [1]. Among bee species, *Apis mellifera* is responsible for most commercially available propolis worldwide [2]. The chemical composition of propolis is highly complex and directly influenced by several factors, particularly botanical source, geographic origin, climate, and seasonal availability of resin-producing plants [3]. Consequently, substantial variations in physicochemical properties and biological activities are commonly observed among propolis samples from different regions [2,3,4,5].

Due to its rich composition in phenolic acids, flavonoids, terpenoids, and prenylated phenylpropanoids, propolis has attracted considerable scientific and commercial interest [6]. Numerous biological properties have been described, including antioxidant, antimicrobial, anti-inflammatory, antiviral, immunomodulatory, and antitumoral activities [7,8,9,10]. These properties have supported the increasing use of propolis in pharmaceutical, cosmetic, nutraceutical, and food formulations, reinforcing the growing global demand for standardized and chemically characterized bee products [6].

Brazil is recognized as one of the major global producers of propolis, exhibiting remarkable diversity in propolis types due to its extensive biodiversity and climatic heterogeneity. Approximately twelve chemically distinct groups of Brazilian propolis have been described to date [11]. Among them, Brazilian green propolis is the most extensively studied and commercially valued type, particularly in Asian markets such as Japan [12]. This propolis is predominantly associated with the plant *Baccharis dracunculifolia* (Asteraceae), which constitutes the principal botanical source used by *A. mellifera* bees for resin collection [2,13]. Green propolis is characterized by high diversity of active compounds, especially artepillin C, caffeoylquinic acid derivatives, flavonoids, and other phenolic constituents associated with important pharmacological activities [3,8].

The state of Minas Gerais is the leading producer of Brazilian propolis, accounting for approximately 70% of national production [12]. Within this state, the Zona da Mata region represents an important area for apiculture and propolis production. Officially classified as part of the Intermediate Geographic Region (IGR) of Minas Gerais, the Zona da Mata comprises 142 municipalities, including Juiz de Fora, Ubá, Viçosa, Ponte Nova, and Cataguases [14]. The region is characterized by mountainous topography and is predominantly located within the Atlantic Forest biome, although transitional vegetation associated with the Cerrado biome is also present [15]. This ecological heterogeneity may directly influence the diversity of resin-source plants available to bees and, consequently, the chemical composition of locally produced propolis [1,3,16].

Beekeeping activities in the Zona da Mata have expanded considerably over recent decades. According to the Association of Beekeepers of Juiz de Fora and Region (APIJUR), the region currently hosts more than 350 beekeepers and meliponiculturists [17,18]. Despite the economic importance of local apiculture, propolis produced in the vicinity of Juiz de Fora remains poorly characterized compared with samples from traditional Cerrado regions of Minas Gerais [1,12]. Although previous studies addressing the influence of seasonality on propolis, the results remain heterogeneous and context-dependent [19,20,21,22,23]. Given the limited information available on propolis from the Zona da Mata region, investigating whether differences in chemical profiles between collection periods are associated with antioxidant, antimicrobial, and antitumor activities may contribute to a better understanding of regional propolis variability and provide useful information for future standardization and quality control strategies [4].

Therefore, the present study aimed to compare the physicochemical characteristics, chemical profiles, and biological activities of propolis samples collected from four apiaries in the Juiz de Fora, Zona da Mata region of Minas Gerais during two distinct seasonal sampling periods in 2023. For this purpose, eight propolis samples collected during summer and winter were characterized in terms of dry extract yield, wax content, total phenolic and flavonoid content, chemical composition by UHPLC-ESI-MS/MS, and electrical properties by electrical impedance spectroscopy. Their biological properties were further evaluated using complementary antioxidant, antibacterial, and cytotoxicity assays. This integrated approach was employed to identify differences between the two sampling periods and to explore their associations with the chemical, physicochemical, and biological profiles of propolis produced in this region.

## 2. Results

### 2.1. UHPLC-ESI-MS/MS Analysis of Propolis Extracts

The annotated compounds are shown in Table 1 and Figure 1, while the chromatograms of the propolis extracts and MS/MS spectra of the annotated compounds are provided in the Supplementary Materials (Supplementary Figures S1–S25).

Overall, qualitative UHPLC-ESI-MS/MS analysis in negative ionization mode revealed a chemically diverse, but qualitatively similar profile among the propolis samples collected in the Zona da Mata, region of Minas Gerais. Seventeen compounds were annotated based on accurate mass measurements, molecular formula, MS/MS fragmentation patterns, and comparison with previously reported mass spectrometric data (Table 1 and Figures S1–S25). The annotated metabolites comprised mono-, di-, and tricaffeoylquinic acid derivatives, hydroxycinnamic acids and their prenylated derivatives, flavonoids, and other phenolic constituents commonly reported in Brazilian propolis. Most annotated compounds were detected in samples from both seasons, indicating substantial similarity in their qualitative chemical profiles despite differences in total phenolic content and other physicochemical parameters.

### 2.2. Characterization of Raw Propolis Samples

The mean dry extract content was higher in propolis samples collected during the summer (572.8 ± 13 mg dry extract/g raw propolis) than in those collected during the winter (493.9 ± 16 mg dry extract/g raw propolis). Conversely, the mean wax content was more than two-fold higher in winter samples (3.2 ± 2.0%) than in summer samples (1.4 ± 0.6%) (Supplementary Table S1). Among the individual samples, summer propolis A1 and B1 exhibited the highest dry extract content, with values of 642.7 ± 13 and 730.2 ± 13 mg dry extract/g raw propolis, respectively, while also showing the lowest wax content (0.61 ± 0.6% and 1.33 ± 0.6%, respectively) (Supplementary Table S1).

Also, total phenolic content (TPC) exhibited marked variation between the two seasons (Figure 2). Summer samples exhibited a TPC values of 166.51 ± 4.0 mg (B1), 193.21 ± 4.2 (C1), 218.38 ± 2.8 (D1) and 260.33 ± 3.8 (A1) mg GAE/g dry extract, whereas substantially lower values were observed in the winter samples, ranging from 85.62 ± 2.8 mg GAE/g dry extract in D2 to 120.75 ± 4.2 mg GAE/g dry extract in A2 (Figure 2). The magnitude of the differences between the two collection periods varied among apiaries. In contrast to TPC, total flavonoid content (TFC) remained comparatively stable across the samples, with an overall mean value of 24.5 ± 2.17 mg QE/g dry extract (Figure 2).

### 2.3. Antioxidant Activity

The DPPH radical scavenging assay revealed marked differences in antioxidant activity among the evaluated samples (Figure 3A). Samples collected during summer generally exhibited greater antioxidant activity, with IC_50_ values ranging from 22.2 ± 0.15 µg/mL for A1 to 54.7 ± 0.18 µg/mL for C1. Overall, higher IC_50_ values were observed for the corresponding winter-collected samples. A similar pattern between the two collection periods was observed in the nitric oxide (NO) scavenging assay.

All evaluated extracts exhibited concentration-dependent NO scavenging activity, with particularly pronounced effects observed for the summer samples. Summer extracts from apiaries A, B, C and D exhibited IC_50_ values of 22.65 ± 2.75, 9.31 ± 0.88, 109.31 ± 0.88 and 37.66 ± 1.66 µg/mL, respectively. On the other hand, winter extracts from apiaries A, B, C and D showed IC_50_ values of >250, 18.54 ± 1.74, 39.38 ± 3.06 and 122.81 ± 30.59 µg/mL, respectively (Figure 3B). Notably, summer extracts from propolis samples A1, B1, and D1, displayed lower IC_50_ values, indicating potent nitric oxide scavenging capacity under the same tested conditions (Figure 3B).

All evaluated extracts exhibited high hydroxyl radical scavenging activity (Figure 3C). Summer extracts from apiaries A, B, C and D showed inhibition percentage of 76.99 ± 3.60%, 76.91 ± 2.35%, 75.56 ± 1.29% and 75.56 ± 1.40%, respectively. Winter extracts from apiaries A, B, C and D showed inhibition percentage of 79.12 ± 4.70%, 77.06 ± 2.03%, 74.39 ± 2.43% and 75.27 ± 1.62%, respectively (Figure 3C). Overall, hydroxyl radical scavenging activity was comparable across samples from the two collection periods (Figure 3C). While DPPH and nitric oxide (NO) assays were selected to construct complete concentration–response curves and calculate the IC_50_ values, the hydroxyl radical assay was designed as a fixed-dose comparative screening step to confirm whether the regional propolis extracts possessed scavenging capacity against this highly aggressive reactive oxygen species. As can be observed, 20 µg/mL proved to be an effective concentration to demonstrate robust activity across all extracts without reaching a floor or plateau effect.

The oxidation curves obtained in the ORAC assay further demonstrated the antioxidant potential of the propolis extracts, particularly those collected during the summer from apiaries A and B, which exhibited inhibition values of 77.9 ± 2.45% and 92.77 ± 7.22%, respectively, at a concentration of 5 µg/mL after 80 min (Figure 4A). The extract from apiary B showed an oxidation profile comparable to that of Trolox (96.07 ± 10.22%), with sustained antioxidant activity for up to 80 min. The summer extracts C1 and D1 showed 58.22 ± 3.47% and 59.72 ± 3.71% of inhibition values, respectively. The winter extracts A2, B2, C2 and D2 exhibited inhibition percentage of 70.17 ± 0.79%, 56.59 ± 7.47%, 57.79 ± 5.14% and 62.92 ± 1.35%, respectively (Figure 4A).

In the *β*-carotene–linoleic acid emulsion assay, all propolis extracts similarly inhibited oxidative degradation and exhibited high protective effects during the first 30 min of the reaction (Figure 4B). Winter samples B2 and D2 maintained the highest inhibition percentages throughout the assay. However, no statistically significant differences in inhibition were observed among the samples after 120 min (*p* > 0.05).

### 2.4. Electrical Impedance Spectroscopy

The impedance spectrum exhibited characteristic profiles associated with dielectric dispersion and relaxation processes in the analyzed samples (Figure 5). Overall, propolis samples collected during the summer showed lower impedance than their corresponding winter samples. At 20 kHz frequency (Supplementary Table S2), summer samples showed a mean impedance of 13.63 ± 1.4 kΩ, while winter samples showed 18.31 ± 1.9 kΩ. Among them, summer samples A1 (11.28 kΩ) and B1 (14.36 kΩ) exhibited the lowest impedance values (Figure 5).

### 2.5. Antimicrobial Activity

The broth microdilution assay showed inhibitory activity against *S. aureus* for all evaluated propolis extracts (Table 2). MIC values were generally comparable between the corresponding summer- and winter-collected samples. Extracts from apiary D exhibited the highest antibacterial activity, with MIC values of 250 µg/mL, whereas most of the remaining samples exhibited MIC values of 500 µg/mL. The only exception was the winter sample B2, which showed lower antibacterial activity, with an MIC > 1000 µg/mL.

None of the evaluated propolis extracts exhibited bactericidal activity at the concentrations tested, as determined by the MBC assay (Table 2). Thus, under the experimental conditions employed, the antibacterial effect of the extracts was predominantly bacteriostatic, characterized by inhibition of bacterial growth without detectable bactericidal activity.

### 2.6. Cytotoxicity Against Tumor and Non-Tumor Cells

The cytotoxic potential of the propolis samples was evaluated against five human tumor cell lines (luminal breast cancer, MCF7; triple-negative breast cancer, MDA-MB-231; melanoma, A-375; hepatoma, HepG2; and glioblastoma, U-87). To determine selectivity, two non-tumor cell lines were included: MCF10A (breast) and PNT2 (prostate). The MTT assay was conducted over 48 h, and the 50% inhibitory concentration (IC_50_) and selectivity indices (SIs) were calculated (Table 3).

To MTT assay, the summer propolis extract from apiary D (D1) exhibited the highest cytotoxic activity among the evaluated samples, with an IC_50_ of 4.00 µg/mL against MDA-MB-231 cells and SI values of 7.86 and 61.72 relative to MCF10A and PNT2 cells, respectively. The other propolis extracts also exhibited SI values > 2.0 against MDA-MB-231 cells when PNT2 cells were used as the non-tumorigenic control, indicating preferential cytotoxicity toward the triple-negative breast cancer cell line (Table 3). Cytotoxic activity was also observed against HepG2 cells, with IC_50_ values reaching from 43.54 µg/mL for D2 to 156.1 µg/mL for B1. Among the extracts evaluated against HepG2 cells, B2, C1, and D1 exhibited SI values > 2.0 (Table 3). As expected, doxorubicin (DOX), used as the positive control, exhibited lower IC_50_ values than the propolis extracts. DOX showed selective cytotoxicity toward the luminal breast cancer, melanoma, and hepatoma cell lines, whereas SI values > 2.0 were not observed for the triple-negative breast cancer or glioblastoma cell lines (Table 3).

Cytotoxicity was also evaluated using the sulforhodamine B (SRB) assay, and IC_50_ and SI values were determined after 48 h of treatment (Table 4). Compared with the MTT assay, the SRB assay generally yielded higher IC_50_ values, while several propolis extracts exhibited increased selectivity toward the tumor cell lines evaluated. A particularly pronounced response was observed against MDA-MB-231 cells. Extract D2 exhibited the lowest IC_50_ value against this cell line (2.517 µg/mL), with SI values of 175.9 and 95.35 relative to MCF10A and PNT2 cells, respectively. Extracts B1 and B2 also showed high selectivity toward MDA-MB-231 cells, with SI values > 92.7 and 38.2, respectively, relative to MCF10A cells.

#### Colony Formation and Cell Migration

Since winter extract D2 exhibited the lowest IC_50_ value and the highest selectivity for MDA-MB-231 cells, additional clonogenic and cell migration (wound healing) assays were performed over 48 h at concentrations corresponding to the IC_50_ (2.517 µg/mL) and IC_50_/2 (1.2585 µg/mL). In the clonogenic assay, sample D2 reduced the colony-forming capacity of MDA-MB-231 cells in a concentration-dependent manner (Figure 6A,B). In the cell migration assay, D2 also significantly inhibited wound closure at both 24 and 48 h and at both concentrations, with no recovery of migratory potential observed at the later time point, demonstrating a sustained effect of the extract (Figure 6C,D).

## 3. Discussion

Qualitative UHPLC-ESI-MS/MS profiling revealed the chemical complexity of the hydroethanolic extracts obtained from propolis samples collected in the Zona da Mata region of Minas Gerais, Brazil.

**Figure 1:**
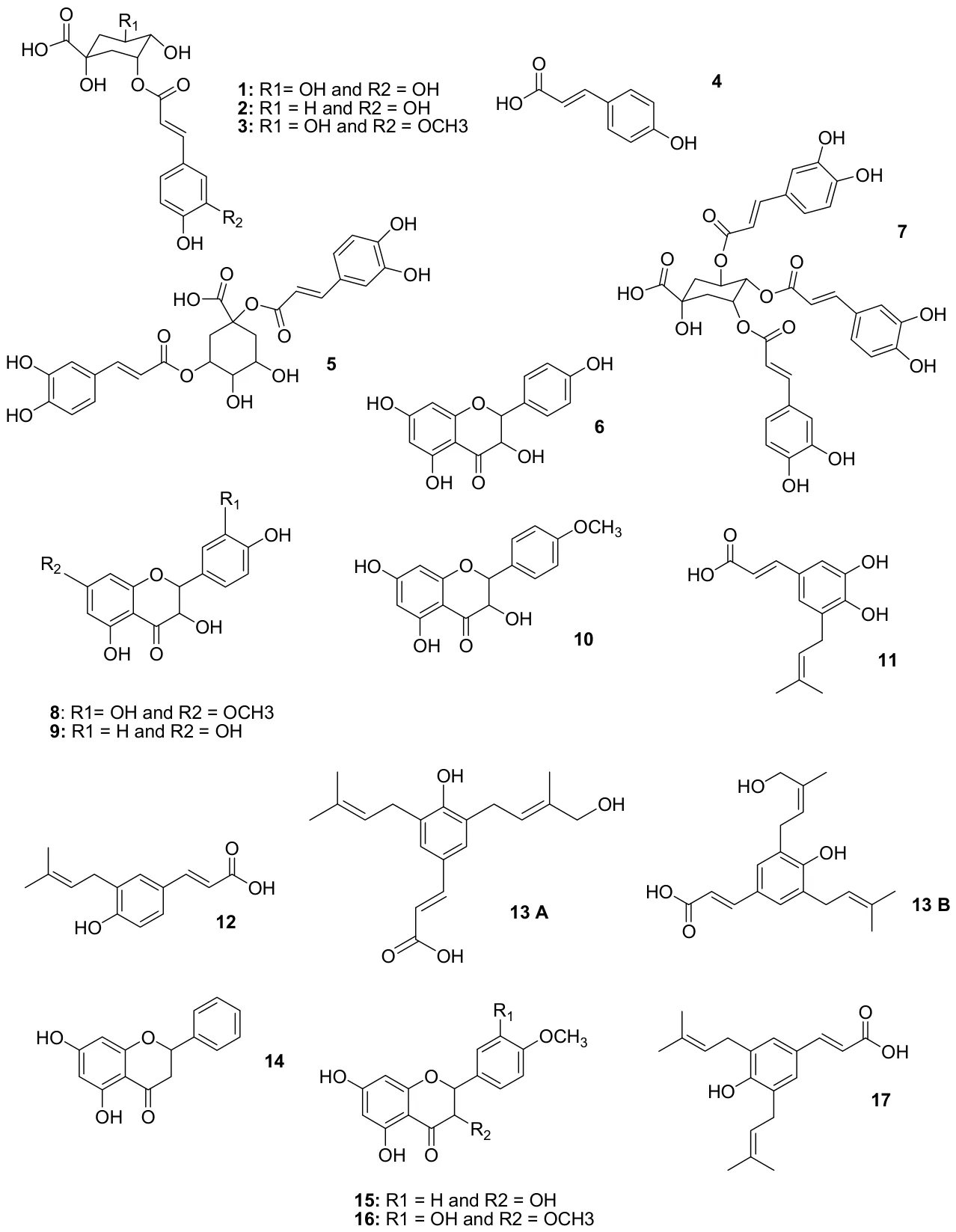
Annotated compounds in propolis extracts from samples produced in the Zona da Mata region of Minas Gerais. Structure **13A** corresponds to Capillartemisin A and **13B** to Capillartemisin B.

**Figure 2:**
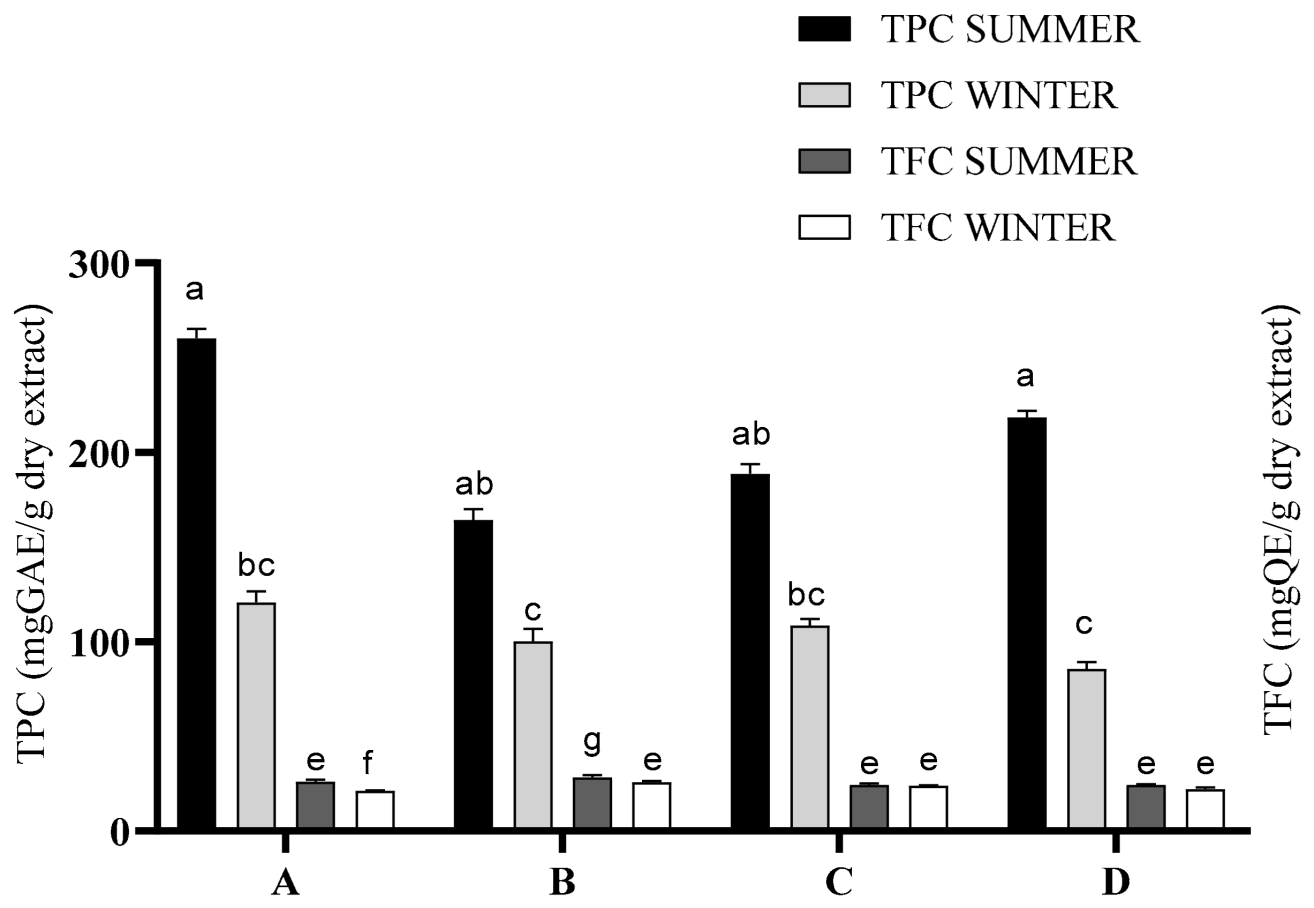
Total Phenolic Content and Total Flavonoid Content in propolis samples from Zona da Mata-MG, harvested in summer and winter seasons. Data are presented as mean ± SD, values showing the same letter did not show significant difference (*p* > 0.05). **A:** Rancho Alegre Apiary. **B:** Belmiro Braga Apiary. **C:** Valadares Apiary. **D:** Salvaterra Apiary.

**Figure 3:**
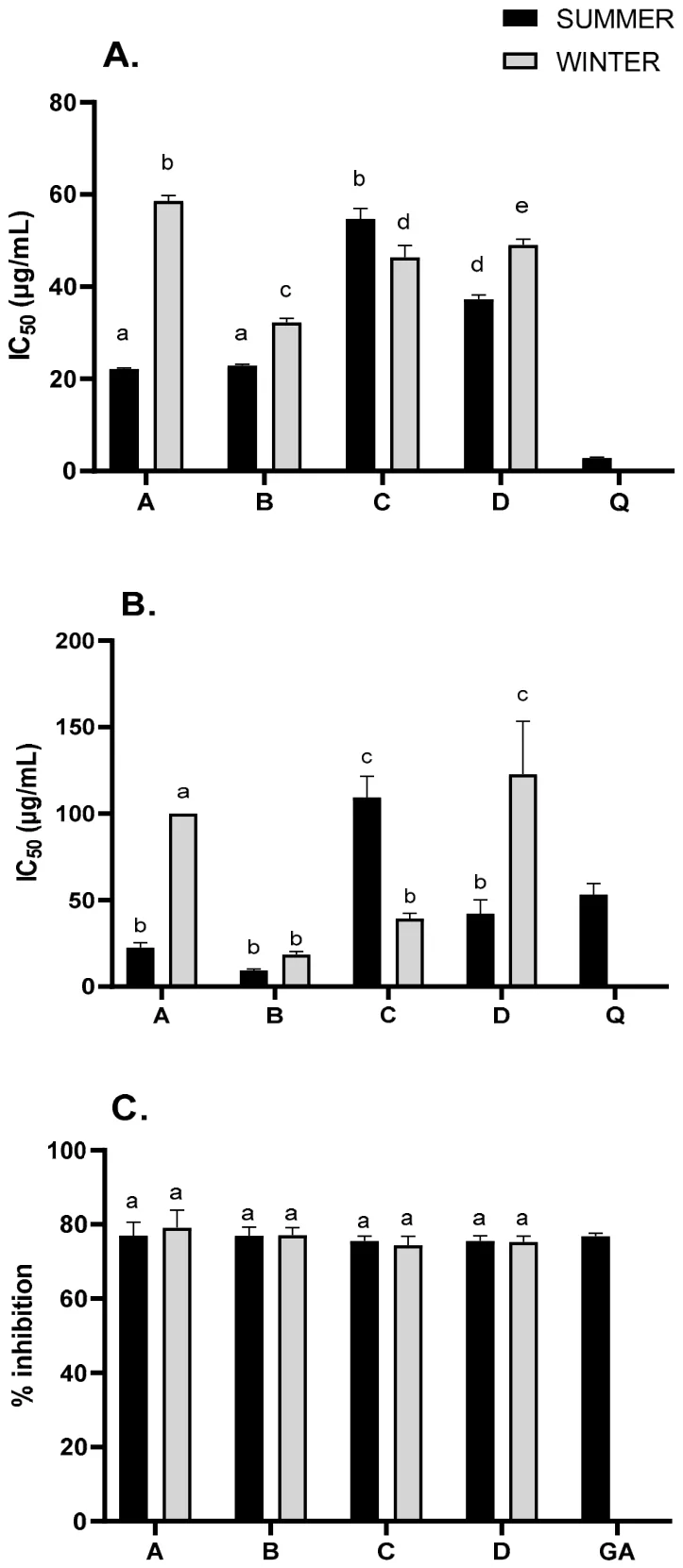
Antioxidant activity of propolis samples collected during summer and winter. Bar graphs represent (**A**) IC_50_ values for 50% inhibition DPPH radical, (**B**) IC_50_ values for 50% inhibition NO radical and (**C**) percentage inhibition OH radical. Data are presented as mean ± SD, values showing the same letter did not show significant difference (*p* > 0.05). **A:** Rancho Alegre Apiary. **B:** Belmiro Braga Apiary. **C:** Valadares Apiary. **D:** Salvaterra Apiary. **Q:** Quercetin. **GA:** Gallic acid.

**Figure 4:**
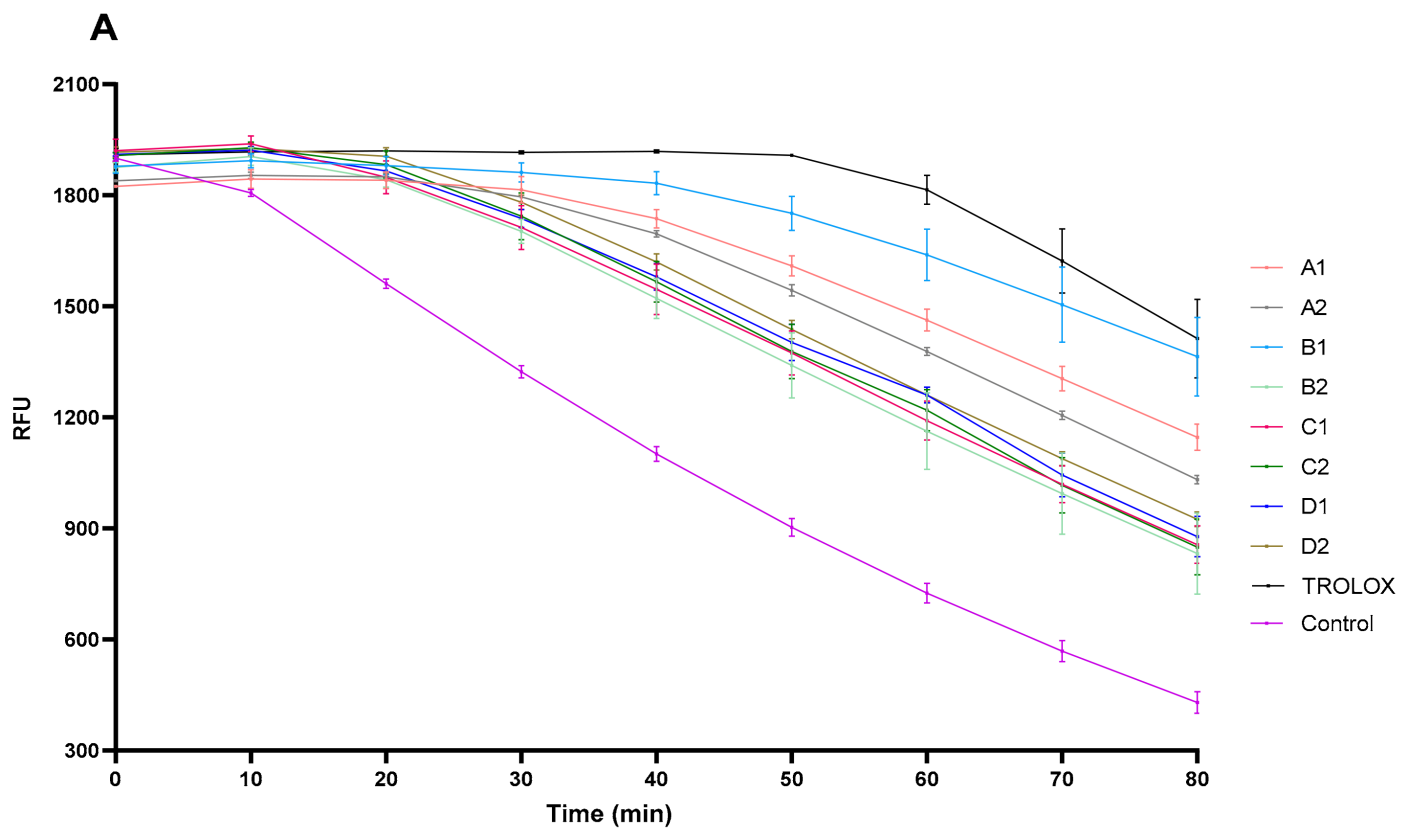

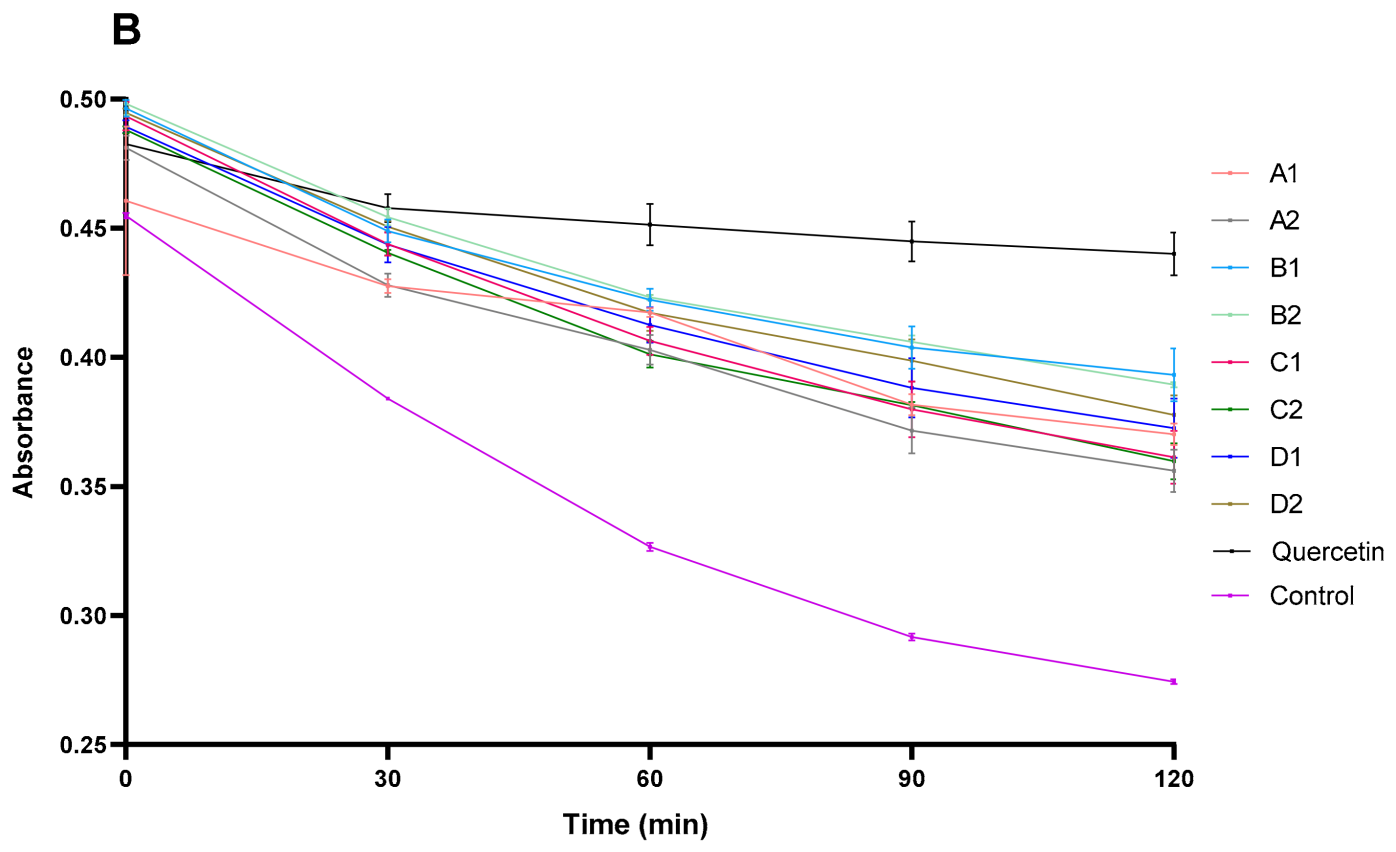
Antioxidant activity of propolis samples collected during summer and winter. (**A**) Peroxyl radical scavenging (ORAC); (**B**) *β*-Carotene bleaching inhibition activity. Experiments were performed in triplicate, and results are presented as mean ± standard deviation. **1**—Samples collected in the summer. **2**—Samples collected in the winter: **A:** Rancho Alegre Apiary. **B:** Belmiro Braga Apiary. **C:** Valadares Apiary. **D:** Salvaterra Apiary. Control (vehicle).

**Figure 5:**
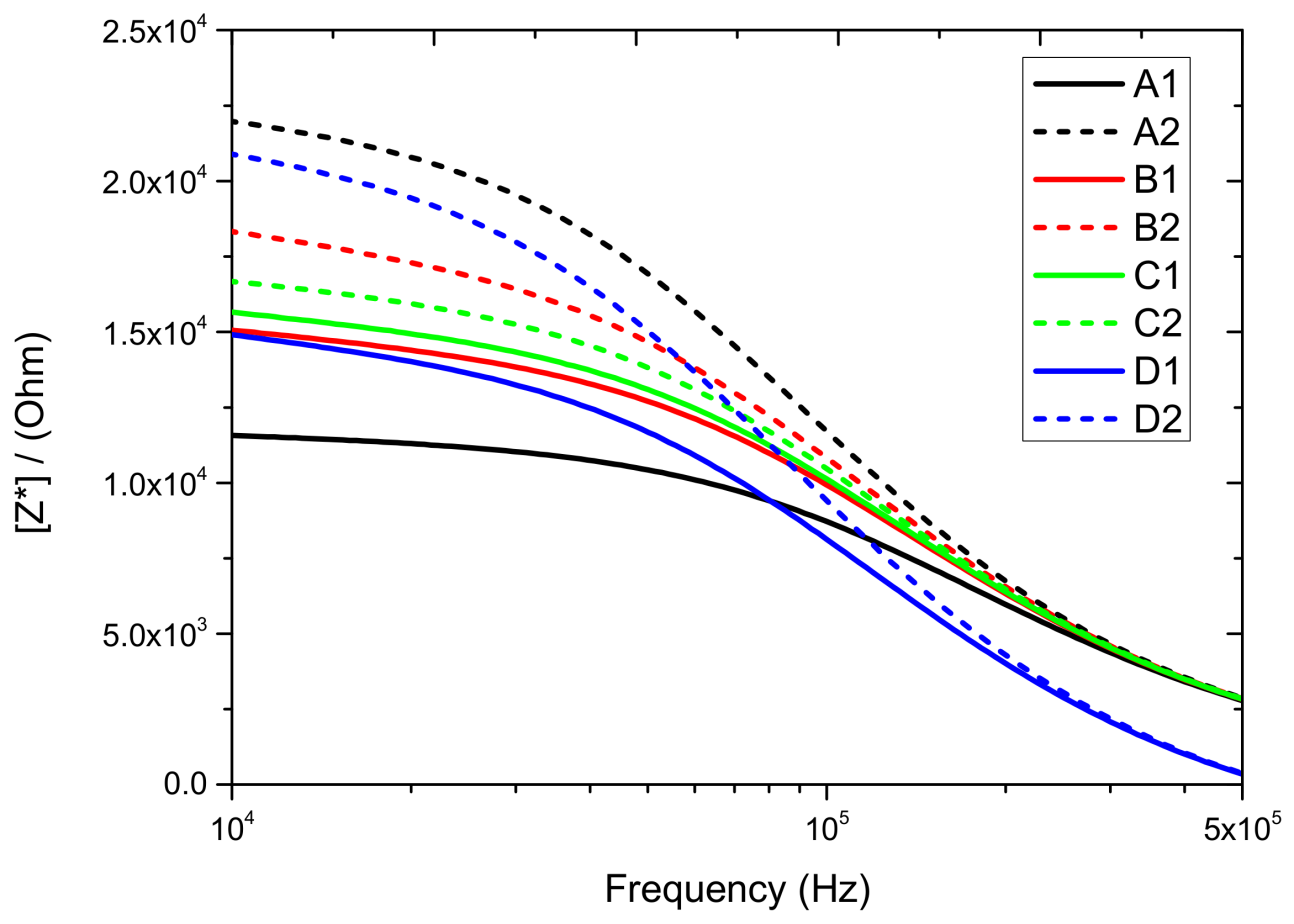
Impedance module (Z) spectrum for propolis samples. **1**—Samples collected in the summer. **2**—Samples collected in the winter. **A:** Rancho Alegre Apiary. **B:** Belmiro Braga Apiary. **C:** Valadares Apiary. **D:** Salvaterra Apiary.

The more polar region of the chromatograms was characterized by several hydroxycinnamoylquinic acid derivatives. Compound **1** (Figure 1), eluting at 4.46 min, displayed a deprotonated molecular ion at *m*/*z* 353.0876, consistent with C_16_H_18_O_9_, and yielded product ions at *m*/*z* 191.0549, 179.0343, 173.0439, and 135.0450. This fragmentation pattern is characteristic of caffeoylquinic acids and supported its annotation as chlorogenic acid. In particular, ions at *m*/*z* 191 and 179 are associated with the quinic acid and caffeic acid moieties, respectively. Additional fragment ions, including *m*/*z* 135 and 173, were also observed and are consistent with previously described fragmentation for caffeoylquinic acids [24,25]. This compound was detected in all eight samples. Compound **2** ([M−H]^−^ at *m*/*z* 337.0930; C_16_H_18_O_8_) produced fragment ions at *m*/*z* 191.0549, 179.0343, and 135.0450 and was annotated as coumaroylquinic acid. In contrast to most hydroxycinnamoylquinic acid derivatives, it was detected only in samples A1, B1, and B2. Compound **3**, annotated as *O*-feruloylquinic acid, exhibited [M−H]^−^ at *m*/*z* 367.1040 and characteristic fragment ions at *m*/*z* 191.0549 and 173.0439 and occurred in all samples. The ion at *m*/*z* 191 is diagnostic of the quinic acid moiety, while the occurrence of *m*/*z* 173 is consistent with fragmentation reported for feruloylquinic acid isomers [24,25].

Compound **4** (Figure 1) showed [M−H]^−^ at *m*/*z* 163.0397 and a major fragment ion at *m*/*z* 119.0494, corresponding to a neutral loss of 44 Da (CO_2_), supporting its annotation as *p*-coumaric acid. This compound was detected in all samples, except summer sample C1 [26].

Compound **5** ([M−H]^−^ at *m*/*z* 515.1183) (Figure 1) generated fragment ions at *m*/*z* 353.0876, 191.0549, 179.0343, and 173.0439 and was annotated as dicaffeoylquinic acid. The fragment at *m*/*z* 353 results from the loss of a caffeoyl moiety, followed by fragmentation characteristic of caffeoylquinic acid. Compound **7**, with [M−H]^−^ at *m*/*z* 677.1535, generated sequential ions at *m*/*z* 515.1183 and 353.0876, followed by *m*/*z* 179.0343 and 173.0439, supporting its annotation as tricaffeoylquinic acid. Dicaffeoylquinic acid occurred in all eight samples, whereas tricaffeoylquinic acid was absent only from summer sample D1.

Several flavonoids were also annotated. Compound **6** ([M−H]^−^ at *m*/*z* 287.0559; C_15_H_12_O_6_) (Figure 1) generated fragment ions at *m*/*z* 259.0610 and 125.0249 and was assigned as aromadendrin. Compound **8** ([M−H]^−^ at *m*/*z* 315.0501) yielded *m*/*z* 300.0264, 271.0226, and 165.9904 and was annotated as rhamnetin. The formation of *m*/*z* 300 is consistent with the loss of a methyl group from a methoxylated flavonol, whereas lower-mass fragments are consistent with subsequent fragmentation of the flavonoid skeleton. Both compounds were detected in all samples.

Compound **9** exhibited [M−H]^−^ at *m*/*z* 285.0412 and fragments at *m*/*z* 255.0281, 179.0343, 161.0232, and 151.0031, supporting its annotation as kaempferol. Compound **10** ([M−H]^−^ at *m*/*z* 301.0731; C_16_H_14_O_6_) generated ions at *m*/*z* 283.0612, 227.0696, 152.0108, 151.0031, and 125.0249 and was assigned as dihydrokaempferide. Both metabolites occurred in all eight propolis samples. Dihydrokaempferide has previously been reported in Brazilian propolis with similar diagnostic fragmentation pattern, including ions at *m*/*z* 283, 227, 152/151, and 125 [25,27].

The flavanone pinocembrin (compound **14**) was annotated from the deprotonated ion at *m*/*z* 255.0674 and fragment ions at *m*/*z* 213.0561 and 151.0031. It was detected in all samples. Two additional methoxylated flavonols were annotated. Compound **15** at *m*/*z* 299.0558 [M−H]^−^ produced ions at *m*/*z* 284.0331, 164.0107, and 151.0031, consistent with kaempferide, with the product at *m*/*z* 284 corresponding to loss of CH_3_ from the methoxy substituent. Compound **16** ([M−H]^−^ at *m*/*z* 329.0658) produced ions at *m*/*z* 314.0446, 299.0558, 271.0259, and 179.0343 and was annotated as quercetin dimethyl ether. Kaempferide was detected in all samples except summer sample C1, whereas quercetin dimethyl ether occurred in all samples.

A second major group comprised prenylated cinnamic acid derivatives characteristic of Brazilian propolis. Compound **11** showed [M−H]^−^ at *m*/*z* 247.0959 and fragments at *m*/*z* 203.1071 and 148.0523 and was annotated as dihydroxyprenylcinnamic acid. Compound **12**, drupanin, displayed [M−H]^−^ at *m*/*z* 231.1030 and diagnostic fragments at *m*/*z* 187.1120 and 132.0577. While compound **11** was absent from C1 and D2, drupanin was detected in all eight samples. Prenylated cinnamic acid derivatives exhibited fragmentation pathways characterized by neutral losses associated with their prenyl side chains [28].

Compound **13** ([M−H]^−^ at *m*/*z* 315.1628; C_19_H_24_O_4_), detected in A1, A2, B1, B2, D1, and D2, generated fragment ions at *m*/*z* 285.1483, 271.1708, 253.1587, 198.1037, and 186.1054 and was tentatively annotated as capillartemisin A/B. Previous analyses of Brazilian green propolis have reported capillartemisin-type constituents with [M−H]^−^ at m/z 315 and fragmentation involving m/z 271 and related fragments [27].

Finally, compound 17, eluting at 10.54 min, showed [M−H]^−^ at *m*/*z* 299.1655 (C_19_H_24_O_3_) and generated the characteristic fragment at *m*/*z* 255.1752, corresponding to a neutral loss of 44 Da. The compound was annotated as artepillin C and was detected in all eight propolis samples. This fragmentation pattern has been previously reported for artepillin C in Brazilian green propolis [29].

The UHPLC-ESI-MS/MS data demonstrated a high degree of qualitative chemical similarity among propolis samples collected from the four apiaries and during the two seasons periods. Of the 17 annotated metabolites, chlorogenic acid, *O*-feruloylquinic acid, dicaffeoylquinic acid, aromadendrin, rhamnetin, kaempferol, dihydrokaempferide, drupanin, pinocembrin, quercetin dimethyl ether, and artepillin C were detected in all eight samples. In contrast, coumaroylquinic acid, dihydroxyprenylcinnamic acid, capillartemisin A/B, *p*-coumaric acid, tricaffeoylquinic acid, and kaempferide exhibited more restricted distributions. Overall, these findings indicate substantial overlap in the qualitative chemical profiles of the eight propolis extracts, accompanied by a smaller subset of metabolites with sample-dependent detection.

The compounds consistently detected across the eight samples included representatives of caffeoylquinic acid derivatives, flavonoids, and prenylated phenylpropanoids, indicating substantial qualitative similarity among the analyzed propolis extracts.

Among the annotated compounds, the consistent detection of artepillin C and drupanin across all samples is particularly noteworthy. These prenylated cinnamic acid derivatives are widely recognized as characteristic constituents of Brazilian green propolis and have been frequently associated with *Baccharis dracunculifolia*, a major resin source reported for this propolis type [13,30]. The detection of *p*-coumaric acid and dihydroxyprenylcinnamic acid in some samples further supports the similarity of their chemical profiles to those previously described for *B. dracunculifolia*. However, these chemical markers alone cannot conclusively establish botanical origin, which would require complementary evidence from approaches such as palynological analysis and/or direct comparison with plant exudates.

In contrast, coumaroylquinic acid was detected only in samples from apiaries A and B, whereas dihydroxyprenylcinnamic acid, capillartemisin A/B, p-coumaric acid, tricaffeoylquinic acid, and kaempferide were absent from one or more samples. These differences indicate some degree of qualitative chemical heterogeneity among the analyzed propolis extracts. However, no consistent collection-period-associated pattern of presence or absence was evident for most of these compounds. Therefore, within the limitations of the qualitative profiling and sampling design, no major shift in the overall chemical profile was evident between the summer- and winter-collected samples.

Despite this overall chemical similarity, the physicochemical characterization of the eight propolis samples revealed marked differences between the two collection periods in dry extract yield, wax content, and total phenolic content, whereas total flavonoid content remained comparatively stable. Overall, summer-collected samples exhibited higher dry extract and total phenolic content and lower wax content than the corresponding winter-collected samples. These collection-period-associated differences may reflect variations in the relative incorporation of resinous and wax components into propolis. Changes in plant resin availability and bee foraging behavior under different environmental conditions represent plausible contributing factors, although these relationships cannot be established from the present sampling design.

Among the evaluated samples, A1 exhibited the highest total phenolic content, followed by D1, both collected during the summer. Interestingly, propolis samples from apiaries A and B displayed macroscopic characteristics commonly associated with Brazilian green propolis, including a greenish coloration, brittle consistency, and relatively low wax content. In contrast, samples from apiaries C and D showed a brownish coloration and a softer, waxier consistency (Figure 7). Such variability in physicochemical characteristics may reflect differences in the relative contribution of plant-derived resinous and wax components, potentially associated with local vegetation, resin availability, and other environmental factors [31].

*B. dracunculifolia*, widely recognized as a major botanical source of Brazilian green propolis, undergoes marked vegetative development and flowering during the rainy season, which extends approximately from December to May in southeastern Brazil [13]. Seasonal changes in plant phenology and resin availability may influence the materials accessible to foraging bees and, consequently, the relative incorporation of resinous and wax components into propolis. During colder and drier periods, changes in the availability of resin-producing plants may likewise modify the relative contribution of these materials, potentially affecting propolis composition and extraction yield [32,33]. In this context, the higher dry extract and total phenolic content and lower wax levels generally observed in the summer-collected samples are consistent with greater availability and incorporation of resinous plant materials during this collection period. However, this interpretation remains tentative because neither plant resin availability nor bee foraging behavior was directly assessed in the present study.

Oxidative and nitrosative stress, resulting from an imbalance between the production of reactive oxygen species (ROS) and reactive nitrogen species (RNS) and cellular antioxidant defenses, plays an important role in the pathophysiology of several chronic disorders, including cancer, diabetes mellitus, neurodegenerative diseases, and cardiovascular disorders [34]. Given the chemical complexity of propolis and the multiple mechanisms through which its constituents may exert antioxidant effects, no single assay can comprehensively characterize its antioxidant potential. Therefore, in the present study, a complementary panel of in vitro assays based on different reaction mechanisms and experimental systems was employed, including nitric oxide (NO) scavenging, DPPH radical scavenging, hydroxyl radical scavenging, oxygen radical absorbance capacity (ORAC), and *β*-carotene-linoleic acid bleaching assays.

Interestingly, the differences in antioxidant responses observed between the two collection periods partially paralleled those in total phenolic content, with summer-collected samples generally exhibiting higher phenolic levels and stronger antioxidant activity in several of the assays employed. The lower IC_50_ values observed for the summer extracts in the DPPH and nitric oxide scavenging assays indicate greater radical scavenging activity. This association was further supported by Spearman’s rank correlation analysis, which revealed a strong negative correlation among total phenolic content and DPPH IC_50_ values (*ρ* = −0.864, *p* < 0.0001), indicating that samples with higher phenolic content required lower concentrations to achieve 50% DPPH radical scavenging. However, given the small number of samples analyzed, the analysis was considered exploratory. This finding is nonetheless consistent with previous studies reporting associations between phenolic content and antioxidant activity in propolis [35,36].

DPPH is a synthetic and relatively stable free radical widely used to assess the radical scavenging activity of compounds and complex mixtures, particularly in organic or hydroalcoholic media [34]. In contrast, the nitric oxide and hydroxyl radical scavenging assays provide complementary information on the ability of propolis constituents to interact with biologically relevant reactive species under different experimental conditions. Nitric oxide (NO) is a reactive nitrogen species involved in several physiological and pathological processes and can react rapidly with superoxide to form peroxynitrite, a highly reactive oxidant capable of damaging proteins, lipids, and nucleic acids [37]. Therefore, the NO scavenging activity observed for the propolis extracts complement the DPPH findings and indicates that their antioxidant properties are not restricted to a single radical system or reaction medium.

Beyond the radical scavenging assays discussed above, the ORAC and *β*-carotene-linoleic acid bleaching assays provided complementary information on antioxidant performance in distinct reaction environments. The ORAC analysis evaluates the ability of antioxidants to inhibit oxidation initiated by peroxyl radicals generated in an aqueous system and is based primarily on a hydrogen atom transfer mechanism [34]. In contrast, the β-carotene-linoleic acid bleaching assay assesses the ability of antioxidants to inhibit the oxidative degradation of β-carotene induced by lipid oxidation products generated from linoleic acid in an emulsion system [38]. This latter assay provides useful information on antioxidant behavior in a heterogeneous lipid-containing environment, in which antioxidant effectiveness depends not only on intrinsic radical scavenging capacity, but also on physicochemical properties such as polarity, solubility, and partitioning at the oil–water interface [38,39]. Thus, differences in the antioxidant responses observed among the assays may reflect the ability of individual propolis constituents to act under distinct chemical and physicochemical environments.

The differences observed between the two collection periods may also be reflected in the electrical properties of the propolis extracts. The impedance profiles observed among the samples may reflect differences in ionic conductivity and in the nature and relative contribution of electroactive constituents bearing ionizable or redox-active functional groups [40,41]. In this context, the lower impedance values observed for the summer-collected samples may be associated with physicochemical and compositional differences that favor charge transport at the electrode–solution interface [42]. The frequency of 20 kHz was selected because it corresponds to a region of relatively stable impedance within the characteristic plateau of the spectra, providing a representative measurement point while avoiding the transition region. At 20 kHz frequency Spearman’s rank correlation analysis reveals significant negative correlations between electrical impedance and both total phenolic content (*ρ* = −0.833, *p* < 0.01) and total flavonoid content (*ρ* = −0.714, *p* < 0.05). Interestingly, this pattern partially parallels the antioxidant results, as summer-collected samples generally exhibited higher phenolic content and stronger activity in several antioxidant assays. Although a direct mechanistic relationship between electrical impedance and antioxidant activity cannot be established from the present data, these parallel trends suggest that impedance measurements may provide complementary information on physicochemical differences among propolis extracts collected at different sampling periods [42].

According to commonly used classification criteria for plant-derived extracts [43], the MIC values observed for most of the propolis samples in the current study are consistent with moderate antibacterial activity against *S. aureus*. MIC values ≤ 100 µg/mL have been classified as indicative of strong activity, values between 100 and 625 µg/mL as moderate activity, and values > 625 µg/mL as weak activity [43]. Accordingly, the MIC values of 250–500 µg/mL observed for most samples fall within the moderate activity range, whereas the winter sample B2 (MIC > 1000 µg/mL) exhibited weak activity.

Interestingly, antibacterial activity was generally comparable among the corresponding summer- and winter-collected samples, despite the marked differences in total phenolic content observed between the two collection periods. Total flavonoid content, in contrast, remained comparatively stable across the evaluated samples. Although these parallel patterns do not establish a direct relationship between flavonoid content and antibacterial activity, they raise the possibility that flavonoids, together with other constituents shared across the samples, may contribute to the antibacterial properties of these propolis extracts.

Previous studies support the potential contribution of phenolic constituents to the antibacterial properties of propolis. Da Silva et al. [44] reported negative correlations between the content of flavonoids and hydroxycinnamic acids and MIC values against *S. aureus*, indicating greater antibacterial activity at higher concentrations of these constituents. Similarly, Nichitoi et al. [45] reported that an increased proportion of flavonoids was associated with larger inhibition zones against Gram-positive bacteria. In this context, the comparatively stable total flavonoid content observed across the samples from the two collection periods may partially contribute to the generally similar antibacterial activity observed in the present study. However, this association remains hypothetical, as total flavonoid measurements do not allow the contribution of individual compounds or potential interactions among constituents to be established.

Previous studies have reported heterogeneous patterns regarding temporal and seasonal variation in the antibacterial activity of propolis. Jorge et al. [8] reported substantial temporal variation in prenylated *p*-coumaric acid derivatives without corresponding changes in MIC values. On the other hand, Bueno-Silva et al. [46] observed greater antibacterial activity against *S. aureus* in samples collected between July and November, coinciding with higher concentrations of the isoflavonoids, neovestitol and vestitol. These contrasting findings suggest that temporal differences in propolis antibacterial activity cannot be explained solely by variations in broad chemical classes and may depend on the abundance and combination of specific bioactive constituents.

Overall, the antibacterial activity of propolis likely results from the combined contribution of multiple constituents rather than from a single chemical class, potentially involving additive or other interactions among bioactive compounds [22,47]. In the present study, the marked differences in total phenolic content between the two collection periods contrasted with the comparatively stable total flavonoid content and antibacterial activity. However, because the UHPLC-ESI-MS/MS analysis provided qualitative chemical profiling rather than targeted quantification, the contribution of individual metabolites to these biological responses cannot be established from the current dataset. Future targeted quantitative analyses of selected chemical markers may help.

Regarding cytotoxic activity, the propolis extracts exhibited markedly different responses across the tumor cell lines evaluated, reflecting, at least in part, the molecular and metabolic heterogeneity of solid tumors [48]. Among the evaluated samples in the MTT assay, the summer propolis D1 was particularly significant, exhibiting the highest cytotoxic activity against MDA-MB-231 cells, with an IC_50_ of 4.00 µg/mL and SI values of 7.86 and 61.72 relative to MCF10A and PNT2 cells, respectively. Triple-negative breast cancer (TNBC) is a highly heterogeneous and biologically aggressive breast cancer subtype associated with an increased risk of recurrence and metastasis and poorer clinical outcomes [49,50,51]. The absence of progesterone receptor, estrogen receptor, and human epidermal growth factor receptor 2 (HER2) expression precludes the use of conventional endocrine and HER2-targeted therapies, while resistance to available systemic treatments remains a major therapeutic challenge [49,50,51]. Although cytotoxic effects of propolis and propolis-derived constituents against TNBC models have previously been reported [52,53], the pronounced activity of D1 and D2 propolis extracts, combined with its preferential cytotoxicity toward MDA-MB-231 cells, highlights this propolis as the most promising identified in the present study. Particularly relevant is its SI of 7.86 relative to MCF10A cells, since this non-tumorigenic mammary epithelial cell line provides a tissue-related reference for evaluating selectivity toward breast cancer cells.

The propolis extracts also exhibited cytotoxic activity against HepG2 cells in the MTT assay, with IC_50_ values ranging from 43.54 µg/mL for D2 to 156.1 µg/mL for B1, while B2, C1, and D1 exhibited SI values > 2.0. Previous studies have demonstrated that propolis can reduce HepG2 cell viability and induce apoptosis through mechanisms involving cellular stress responses and activation of apoptotic pathways, including ATF3 and caspases 3 and 9 [54]. Nevertheless, because these molecular mechanisms were not investigated in the present study, the observed effects should be interpreted primarily as evidence of cytotoxic activity rather than as demonstration of a specific apoptotic mechanism. Similarly, although HepG2 cells are widely used in studies of oxidative stress and the MTT assay reflects cellular metabolic activity [55,56], these findings alone do not demonstrate modulation of the intracellular redox state. The remaining tumor cell lines were less responsive to the propolis extracts, generally exhibiting IC_50_ values > 70 µg/mL and limited selectivity.

**Figure 6:**
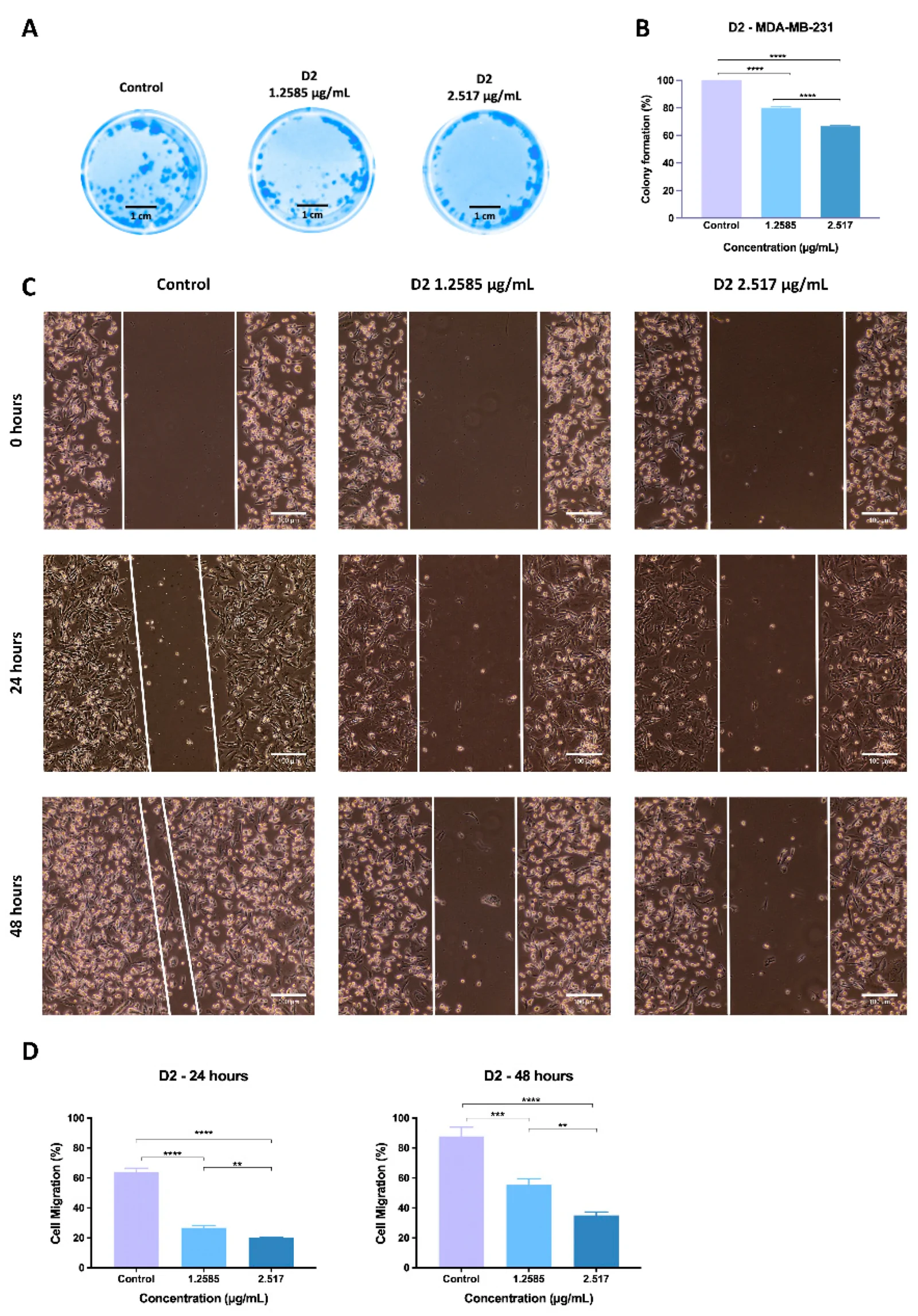
Effects of the winter propolis extract D2 on the clonogenicity and migration of triple-negative breast cancer cells (MDA-MB-231). (**A**) Representative images of wells and formed colonies. (**B**) Percentage of colonies formed after 48 h of treatment with D2 at concentrations of 2.517 and 1.2585 µg/mL. (**C**) Representative images of cell migration. (**D**) Quantification of cell migration after treatment with D2 for 0, 24, and 48 h. Experiments were performed in triplicate, and results are presented as mean ± standard deviation. Statistical significance was determined by ANOVA followed by Tukey’s post hoc test (** *p* < 0.01; *** *p* < 0.0001; **** *p* < 0.00001).

**Figure 7:**
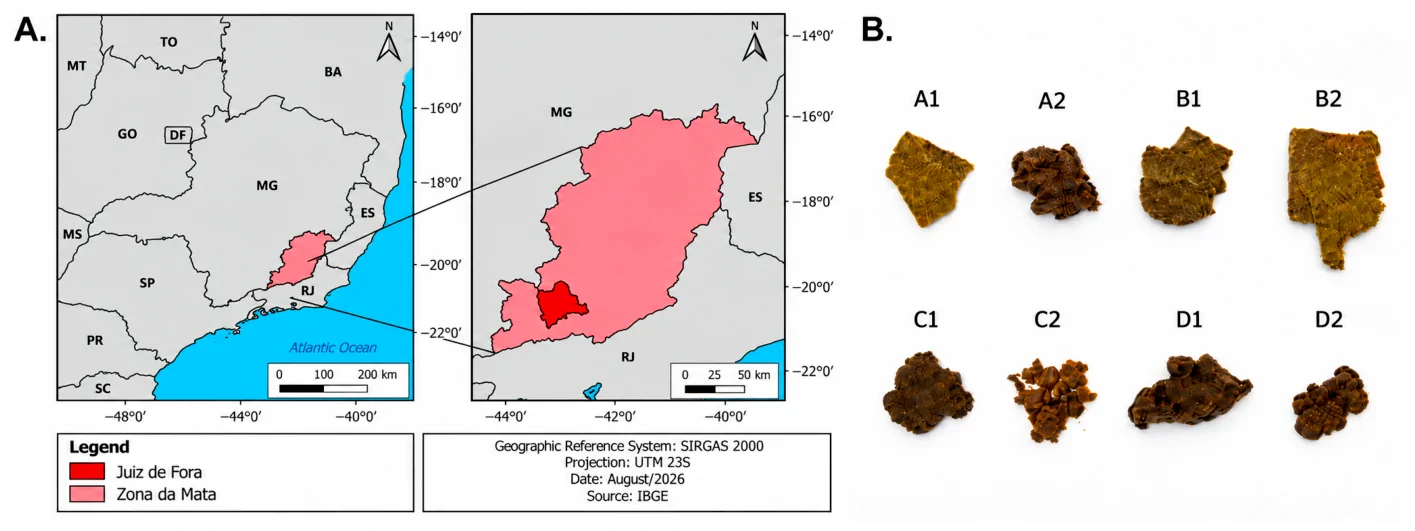
(**A**) Brazilian political map, with emphasis on the studied region traditionally knowledge as Zona da Mata-MG. Source: IBGE. (**B**) Propolis samples collected in Zona da Mata region. **1**—Samples collected in the summer. **2**—Samples collected in the winter. **A:** Rancho Alegre Apiary. **B:** Belmiro Braga Apiary. **C:** Valadares Apiary. **D:** Salvaterra Apiary.

The results obtained from the SRB assay confirmed the cytotoxicity of the analyzed samples, despite the profile differing from that of the MTT assay. The two assays evaluate different aspects: MTT reflects mitochondrial metabolic activity, whereas SRB estimates total protein mass, which correlates with the number of viable cells. While sample D1 showed the most pronounced selectivity for the MDA-MB-231 cell line in the MTT assay, all samples demonstrated significant selectivity in the SRB assay, with B1 and D2 standing out. This finding was confirmed by clonogenic and migration assays performed on MDA-MB-231 cells using sample D2 (which showed the highest selectivity in the SRB assay) (Figure 6). The clonogenic assay, by assessing the cells’ ability to survive and form colonies following treatment, provides a measure of the antiproliferative effect over time rather than over a short period [57]. Migration, in turn, is particularly relevant in the context of TNBC, as it is associated with metastatic potential [58]. Sample D2 significantly inhibited both mechanisms.

Interestingly, the cytotoxicity results did not parallel the patterns observed for total phenolic content and antioxidant activity across the two collection periods. Summer-collected samples generally exhibited higher phenolic content and stronger antioxidant responses in several assays, whereas cytotoxic activity showed no consistent collection-period-associated pattern and varied considerably among the cell lines and viability assays. Notably, D1 and D2, collected from the same apiary during summer and winter, respectively, exhibited pronounced cytotoxic activity and selectivity toward MDA-MB-231 cells, although their relative responses differed between the MTT and SRB assays. These findings indicate that the cytotoxic effects of the propolis extracts cannot be explained solely by total phenolic content or antioxidant capacity. Instead, the activity of D1 and D2 may reflect differences in their specific chemical composition and interactions among bioactive constituents within the complex propolis matrix. Considerable variation in the cytotoxic potency and selectivity of propolis from different geographical and botanical origins has previously been associated with differences in chemical composition [59,60]. In the present study, the distinct responses among extracts, including samples obtained from the same apiary at different collection periods, further highlight the chemical and biological complexity of propolis. However, because the UHPLC-ESI-MS/MS analysis was qualitative, the contribution of individual metabolites or quantitative compositional differences to cytotoxic activity cannot be established. Targeted quantitative characterization of selected metabolites in D1 and D2 would therefore be valuable in future studies to identify constituents potentially associated with their pronounced activity against MDA-MB-231 cells.

## 4. Materials and Methods

### 4.1. Chemicals and Reagents

The DPPH, sodium nitroprusside—SNP, sulfanilamide, N-(naphthyl) ethylenediamine—NED, 2,2′-azobis2-amidino-propane dihydrochloride, quercetin, chloramphenicol, gallic acid and dimethyl sulfoxide (DMSO) were obtained from Sigma-Aldrich^®^ (St. Louis, MO, USA). The phosphoric acid was purchased from Quimibrás Indústrias Químicas (Cubatão, Brazil). Fluorescein and Trolox, methanol and acetonitrile to both analyses and assays were acquired from Merck^®^ Company Inc. (Darmstadt, Germany). Formic acid and acetic acid of HPLC grade were provided by Aladdin Industrial Corporation^®^ (Shanghai, China). UHPLC-MS-grade water was prepared by the Milli-Q water purification system from Millipore^®^ (Bedford, MA, USA).

### 4.2. Propolis Sample Collection and Preparation

The sampling procedures were conducted in accordance with Brazilian legislation regarding access to genetic heritage and associated traditional knowledge (Law No. 13, 123/2015), under registration number A8BB773. Raw propolis samples were kindly provided by beekeepers associated with the Association of Beekeepers of Juiz de Fora and Region.

Propolis samples were collected from four apiaries located in the Zona da Mata region of Minas Gerais, Brazil, at two sampling points in 2023, in March (summer) and September (winter). One raw propolis sample was obtained from each apiary at each sampling point, resulting in eight field samples (four apiaries × two collection periods). Samples were coded according to apiary and collection period: A1, B1, C1, and D1 corresponded to samples collected in summer, whereas A2, B2, C2, and D2 corresponded to samples collected in winter. Apiaries A, B, C, and D were located in Rancho Alegre, Belmiro Braga, Valadares, and Salvaterra, respectively.

For each of the eight raw propolis samples, three separate 1.0 g portions were independently processed through the complete extraction procedure, resulting in three independently prepared extracts per field sample. Each portion was separately pulverized using a porcelain mortar and pestle, transferred to an amber flask, and macerated with 30 mL of 70% hydroethanolic solution for 24 h at room temperature under protection from light. After filtration, the remaining residue was subjected to ultrasound-assisted extraction for 30 min using fresh hydroethanolic solvent under the same conditions. The resulting extracts were filtered and concentrated under reduced pressure at 50 °C using a rotary evaporator [61]. The dry extracts were weighed and stored under refrigeration until analysis. Extraction yield was expressed as milligrams of dry extract per gram of raw propolis (mg dry extract/g raw propolis).

### 4.3. Chemical Characterization by UHPLC-ESI-MS/MS

Chemical characterization of the propolis extracts was achieved using an ultra-high-performance liquid chromatography system coupled to a quadrupole time-of-flight mass spectrometer (UHPLC-QTOF-MS/MS) fitted with an electrospray ionization (ESI) source (XEVO G2S QTOF, Waters, Milford, MA, USA). Separation of compounds was realized on a BEH C18 column (100 × 2.1 mm, i.d, Waters, Milford, MA, USA), (1.7 µm). The UHPLC-ESI-MS/MS analysis was performed for qualitative chemical profiling and compound annotation.

A binary mobile phase was employed, composed of LC-MS-grade water acidified with 0.1% formic acid (solvent A) and LC-MS-grade acetonitrile (solvent B), carried at a constant flow rate of 0.4 mL/min. The following gradient profile was applied: 5% B from 0 to 2 min, increasing to 98% B between 2 and 14 min, held at 98% B from 14 to 16 min, and returning to 5% B from 16 to 20 min [62,63].

Mass spectra were acquired in negative ion mode, scanning across an m/z window of 100–1000, with the capillary voltage set to 3.5 kV. Collision energy was fixed at 3.5 eV during low-energy scans and ramped between 15 and 30 eV for high-energy acquisitions. The ion source was kept at 120 °C, while the desolvation was set to 500 °C. Nitrogen served both as the desolvation gas, supplied at 800 L/h, and as the cone gas, at flow of 50 L/h.

Mass accuracy throughout the run was monitored via continuous infusion of a leucine enkephalin (*m*/*z* 565.2771) prepared in acetonitrile/formic acid solution (50:50, *v*/*v*) and used as an external lock mass standard. Chromatographic and spectra data were acquired and processed using MassLynx™ and ChromaLynx™ 4.1 softwares (Waters Corporation), as well as MZmine version 4.7.29. Compound annotation relied on the interpretation of MS and MS/MS fragmentation spectra, cross-referencing with previously published data, and the use of computational tools, including MetFrag (https://msbi.ipb-halle.de/MetFrag accessed on 18 November 2025) and PubChem (https://pubchem.ncbi.nlm.nih.gov accessed on 18 November 2025), such as SIRIUS (https://bio.informatik.uni-jena.de/software/sirius/ accessed on 18 November 2025) [62,63].

### 4.4. Determination of Waxes

Extraction was carried out following Bankova et al. [4] with minor modifications. For each propolis sample, 1 g of raw material was manually ground prior to extraction. Maceration was then performed using 10 mL of ethanol with samples kept at room temperature and protected from light for a period of 7 days. Following the extraction period, the insoluble portion was removed by filtration, and the resulting ethanolic extract was kept overnight in a freezer at −16 °C. The extract was subsequently subjected to a second filtration step using filter paper. Waxy residue retained on the filter paper was then dried in an oven at 45 °C until a constant weight was achieved. Wax content was calculated based on the difference in weight before and after drying (*w*/*w*).

### 4.5. Determination of Total Flavonoid Content

Total flavonoid content (TFC) was determinate by the colorimetric aluminum chloride assay, adapted with minor modifications [64], employing quercetin as the reference standard. Stock solutions of the propolis extracts were first prepared by dissolving the samples in methanol to a concentration of 5 mg/mL. Then, aliquots of the extracts were mixed with methanolic solution of 8% aluminum chloride containing 5% acetic acid. The reaction mixture was kept at room temperature for 30 min, then the absorbance readings were taken at 420 nm on a UV–V is spectrophotometer (Shimadzu UV-1800, Tokyo, Japan). A solution containing aluminum chloride and acetic acid without sample was used as the blank. Flavonoid content was carried out using the calibration curve equation y = 0.0733x − 0.0671 (r^2^ = 0.9926), and results were expressed as milligrams quercetin equivalents (QE)/g of dry extract.

### 4.6. Determination of Total Phenolic Content

Total phenolic content (TPC) was determined by the Folin–Ciocalteu colorimetric method with some adaptations [65], using gallic acid as the reference compound. Briefly, stock solutions of the extracts were prepared in methanol at 10.0 mg/mL, followed by serial dilutions ranging from 0.050 to 0.005 mg/mL. Then propolis extract solution (0.250 mL) was mixed with the Folin–Ciocalteu reagent (0.250 mL), sodium carbonate solution (0.500 mL) (20%, *w*/*v*), and distilled water (4 mL). The reaction mixture was incubated in the dark at room temperature for 30 min, and absorbance was measured at 760 nm using a Shimadzu UV-1800 spectrophotometer (Tokyo, Japan). All measurements were performed in triplicate. Total phenolic content was quantified using the analytical curve *y* = 0.0437*x* + 0.0409 (*r*^2^ = 0.9994), and results were expressed as milligrams of gallic acid equivalents (GAE)/g of dry extract.

### 4.7. DPPH Radical Scavenging Assay

Free radical scavenging capacity of the propolis extracts was assessed by the DPPH assay, following the procedure previously described [26], with slight adaptations. Briefly, stock solutions of the extracts were prepared at a concentration of 0.280 mg/mL in methanol, from which a series of dilutions was obtained, spanning a range of 0.140 to 0.002 mg/mL. In light-protected tubes, 1 mL of the diluted sample was combined with 2 mL of the methanolic DPPH solution, and the mixture was left to react for 30 min at room temperature. Absorbance readings were then taken at 515 nm on a Shimadzu UV-1800 spectrophotometer (Tokyo, Japan), with methanol serving as the blank. Antioxidant activity was expressed as IC_50_ (µg/mL) values, corresponding to the necessary concentration to inhibit 50% of DPPH radicals.

### 4.8. Hydroxyl Radical Scavenging Assay

Hydroxyl radical scavenging activity was determined according to previously described methods [66] with adaptations. The extracts were evaluated at a final concentration of 20 µg/mL, and all analyses were performed in triplicate. Reaction mixtures contained deoxyribose (5 mM), hydrogen peroxide solution (100 mM), ferrous sulfate (6 mM), and the evaluated samples. After incubation at room temperature for 15 min, phosphoric acid and thiobarbituric acid solutions were added, followed by heating at 98 °C for 15 min in a water bath. Phosphate buffer was used as the negative control. The absorbance was measured at 532 nm using 96-well microplates.

### 4.9. Oxygen Radical Absorbance Capacity (ORAC) Assay

The Oxygen Radical Absorbance Capacity (ORAC) was performed according to previously reported procedures [67], using Trolox as the reference antioxidant. In 96-well microplates, 25 µL of propolis extract solution (1 mg/mL) and 150 µL of fluorescein solution (40 nM) were added in triplicate and incubated at 37 °C for 30 min. Subsequently, 25 µL of AAPH solution (153 mM) was added to initiate the reaction. Fluorescence readings were recorded every 10 min for 60 min using excitation and emission wavelengths of 485 and 538 nm, respectively. Antioxidant activity was evaluated at a final concentration of 5 µg/mL and expressed as percentage inhibition relative to the negative control.

### 4.10. Nitric Oxide Scavenging Assay

Nitric oxide scavenging capacity was assessed following a previously described method [68], with gallic acid as the reference standard. Stock solutions of propolis extracts were prepared at concentration of 1 mg/mL. In 96-well microplates, 62.5 µL of sample solution and 62.5 µL of sodium nitroprusside (SNP) solution were combined, after which serial dilutions were performed to yield final concentrations between 0.250 and 0.007 mg/mL. The reaction mixtures were kept under light exposure at room temperature for 1 h. Subsequently, 125 µL of Griess reagent, prepared from 0.1% *N*-(1-naphthyl) ethylenediamine and 1% sulfanilamide in 2.5% phosphoric acid (1:1), was plated. Absorbance was measured at 540 nm using phosphate buffer as the negative control. Assays were conducted in triplicate, and antioxidant activity was expressed as IC_50_ values (µg/mL).

### 4.11. β-Carotene/Linoleic Acid Bleaching Assay

The antioxidant activity of the propolis extracts was also evaluated using the *β*-carotene/linoleic acid bleaching assay with minor modifications [39]. The emulsion system was prepared by mixing 100 µL of *β*-carotene solution (10 mg/mL), 30 µL of linoleic acid, 265 µL of Tween 40, and 500 µL of HPLC-grade dichloromethane. After completing solvent evaporation, 20 mL of oxygen-saturated distilled water was immediately added under vigorous stirring. In 96-well microplates, 10 µL of sample solution (1 mg/mL) and 240 µL of the emulsion were added to each well. Methanol was used as the negative control. Absorbance was measured at 470 nm over 120 min at 45 °C. Antioxidant activity was expressed as percentage inhibition of lipid peroxidation relative to the control.

### 4.12. Electrical Impedance Spectroscopy

Electrical impedance (EI) measurements were carried out using a HIOKI 3170 impedance analyzer HIOKI E.E. Corporation^®^ (Ueda, Japan), in accordance with Campos et al. [69] with slight modifications. An alternating sinusoidal electric field was applied to the samples at a peak-to-peak amplitude of 1 V across frequencies ranging from 10 KHz to 500 KHz. Sample measurements were performed using a parallel-plate platinum electrode (geometric constant K = 1.0 cm^−1^) connected to the instrument. All readings were conducted at a fixed temperature of 25 °C. Electrical impedance [*Z*] was the parameter recorded, and the resulting spectra were processed and analyzed in OriginPro 9.0.0 (64-bit), SR2 b87.

### 4.13. Determination of Minimum Inhibitory Concentration (MIC) and Minimum Bactericidal Concentration (MBC)

The minimum inhibitory concentration (MIC) values of the propolis extracts were verified by the broth microdilution method according to the CLSI M07-A10 guidelines, with minor modifications [70]. Chloramphenicol was used as the reference antibiotic control.

Stock solutions of propolis extracts were prepared at 10 mg/mL using dimethyl sulfoxide (DMSO) as solvent and sterile water as diluent. Chloramphenicol stock solutions were prepared at 1 mg/mL according to CLSI recommendations [71]. Serial two-fold dilutions were prepared in triplicate, resulting in final concentrations ranging from 1000 to 8 µg/mL for propolis extracts and from 500 to 2 µg/mL for chloramphenicol.

MIC determination was carried out in 96-well microplates containing 100 µL of cation-adjusted Mueller–Hinton broth, and 10 µL of a bacterial suspension (*S. aureus* ATCC 6538), previously adjusted to the 0.5 McFarland scale. Microplates were then incubated at 37 °C for 16–20 h under aerobic conditions. To reveal bacterial viability, 20 µL of a 2,3,5-triphenyltetrazolium chloride (TTC) solution (1 mg/mL) was subsequently plated, followed by an incubation period of 30 min. Minimal Inhibitory Concentration was defined as the lowest concentration at which no visible growth was detected.

Minimum bactericidal concentration (MBC) values were determined following the approach outlined by Andrews [72]. Briefly, 10 µL were taken from wells revealing no visible turbidity into Mueller–Hinton agar (MHA), which were subsequently incubated for 16–20 h at 35 ± 2 °C under aerobic conditions. The MBC corresponded to the lowest concentration at which no bacterial colonies developed after subculture. Bacteriostatic versus bactericidal activity was distinguished based on whether growth was, respectively, observed or absent on the MHA plates.

### 4.14. Tumor Cell Assays

#### 4.14.1. Cell Culture

In this study the human cell lines were obtained from the American Type Culture Collection (ATCC) and tested for the absence of mycoplasma contamination prior to the experiments. The following cell lines and culture media were used: (i) MCF10A—non-tumorigenic mammary (DMEM/F12, Gibco); (ii) PNT2—non-tumorigenic prostate (RPMI-1640, Gibco); (iii) MCF7—luminal breast cancer (RPMI-1640, Gibco); (iv) MDA-MB-231—triple-negative breast cancer (Leibovitz’s L-15, Gibco); (v) A-375—melanoma (DMEM High Glucose, Gibco); (vi) HepG2—hepatoma (DMEM Low Glucose, Gibco); and (vii) U-87—glioblastoma (DMEM Low Glucose, Gibco). All media were supplemented with 10% fetal bovine serum (FBS, Gibco) and 1× antibiotic–antimycotic solution (Sigma-Aldrich), containing penicillin (10,000 U/mL) amphotericin B (25 μg/mL) and streptomycin (10 mg/mL). The medium used for culturing the MCF10A cell line was also supplemented with hydrocortisone (0.25 µg/mL), insulin (10 µg/mL) and epidermal growth factor (EGF, 10 ng/mL). Cultures were maintained at 37 °C in an incubator with a humidified atmosphere (Thermo Scientific™ Forma™ Series 3 Water Jacketed CO_2_ Incubator, 184 L) (Asheville, NC, USA) and 5% CO_2_, except for the MDA-MB-231 cell line, which was maintained without CO_2_ supplementation, in accordance with the specifications of the culture medium used. Cell growth was monitored by microscopy, and cells were subcultured periodically until reaching 80–90% confluence.

#### 4.14.2. Cell Viability

Cell viability was determined using a colorimetric assay relying on the reduction of 3-(4,5-dimethylthiazol-2-yl)-2,5-diphenyltetrazolium bromide (MTT). For this purpose, seven cell lines, MDA-MB-231 (1.5 × 10^4^ cells/well), PNT2 (1.0 × 10^4^ cells/well), MCF7 (5.0 × 10^3^ cells/well), MCF10A (1.2 × 10^4^ cells/well), A-375 (1.5 × 10^4^ cells/well), HepG2 (4.0 × 10^3^ cells/well), and U-87 (5.0 × 10^3^ cells/well), were plated in 96-well microplates and kept under suitable culture conditions until adhesion and confluence were reached. The cells were then exposed to each of the eight propolis extracts (A1, B1, C1, D1, A2, B2, C2 and D2) at various concentrations (ranging from 7.81 to 1000 µg/mL) for 48 h. DOX (ranging from 0.1 to 3.25 ng/µL) (Fauldoxo^®^, Libbs Farmacêutica, São Paulo, Brazil) was included as a reference drug for comparison of the observed cytotoxic effects. Groups of treated cell with the diluent dimethyl sulfoxide (DMSO, Sigma-Aldrich) and untreated cell were included. In the end of the exposure period, the MTT reagent was added (final concentration of 0.5 mg/mL) and keep on incubation for 4 h at 37 °C. Afterward, the contents of the wells were removed, and the formazan crystals were solubilized in 200 μL of DMSO. Then, the absorbance was measured using a Multiskan FC microplate reader (Multiskan FC—Thermo Fisher Scientific, Waltham, MA, USA) at 570 nm. Cell viability was expressed as a percentage relative to the control group, which was considered to have 100% viability. To determine the IC_50_, the obtained values were subjected to non-linear regression analysis using the treatment concentrations and the corresponding cell viability values. Where applicable, the Selectivity Index (SI) was assessed as the ratio between the IC_50_ obtained for the non-tumor cell line and that determined for the tumor cell lines; values > 2.0 were considered significant [56].

#### 4.14.3. Cell Viability—SRB Assay

Cytotoxicity was also evaluated using the SRB assay. For this purpose, the cell lines MCF10A (1.2 × 10^4^ cells/well), PNT2 (1.0 × 10^4^ cells/well), MCF7 (5.0 × 10^3^ cells/well), MDA-MB-231 (1.5 × 10^4^ cells/well), A-375 (1.5 × 10^4^ cells/well), HepG2 (4.0 × 10^3^ cells/well), and U-87 (5.0 × 10^3^ cells/well) were seeded in 96-well plates and maintained under appropriate culture conditions to allow for adhesion and confluence. Subsequently, the cells were treated with the eight propolis extracts (A1, B1, C1, D1, A2, B2, C2, and D2) at concentrations ranging from 7.81 to 1000 µg/mL for 48 h. After this period, the monolayer was fixed with 50% (*w*/*v*) trichloroacetic acid and stained with 0.04% (*w*/*v*) SRB (Invitrogen, Waltham, MA, USA). Following successive washes to remove excess dye, the SRB bound to cellular proteins was resolubilized in 10 mM Tris-HCl buffer (pH 10.5). Absorbance readings were taken at 510 nm using a Multiskan FC spectrophotometer (Thermo Fisher Scientific, Waltham, MA, USA). Calculations for viability, IC50, and SI were performed as described for the MTT assay. The D2 extract was selected for subsequent assays due to its higher selectivity for MDA-MB-231 cells.

#### 4.14.4. Clonogenic Assay

MDA-MB-231 cells were seeded in 12-well plates (5 × 10^2^ cells/well) and, after 24 h, treated with D2 at concentrations corresponding to the IC50 (2.517 µg/mL) and IC50/2 (1.2585 µg/mL) for 48 h. Subsequently, the medium was removed, and fresh complete culture medium was added to the wells and replaced every 3 days. After 14 days, the cells were washed with 1× phosphate-buffered saline (PBS), fixed with formaldehyde (4% *v*/*v*), and stained with crystal violet (0.5% *w*/*v*). Colony images were captured using the iBright system (Thermo Fisher Scientific, Waltham, MA, USA). Quantitative analysis was performed after dissolving the crystal violet in 33% (*v*/*v*) acetic acid, followed by absorbance readings at a wavelength of 570 nm (Multiskan FC—Thermo Fisher Scientific, Waltham, MA, USA).

#### 4.14.5. Wound-Healing

MDA-MB-231 cells were seeded in 12-well plates (2 × 10^5^ cells/well) and cultured until they reached approximately 90% confluence. A linear scratch was made in the cell monolayer using a sterile 200 μL pipette tip, creating a cell-free area. Subsequently, dead cells were removed with 1× PBS, and the culture medium was replaced with medium containing D2 at concentrations corresponding to the IC50 (2.517 µg/mL) and IC50/2 (1.2585 µg/mL). Representative images of the scratch area were captured at 0, 24, and 48 h using an EVOS™ XL Core inverted microscope (ThermoFisher) at 10× magnification, across different fields per well. The extent of wound closure was quantified using ImageJ software (v. 1.54g), with cell migration determined as a percentage according to the formula: Scratch Closure (%) = [(Initial Area − Final Area)/Initial Area] × 100.

### 4.15. Statistical Analysis

Statistical analyses were performed using GraphPad Prism software version 5.0 (GraphPad Software Inc., San Diego, CA, USA). Results were expressed as mean ± standard deviation (SD) of three independently prepared extraction replicates (*n* = 3) from each of the eight original propolis samples. Differences among groups were evaluated by one-way analysis of variance (ANOVA), followed by Tukey’s multiple comparison test. Statistical significance was set at *p* < 0.05.

The distributions of the variables considered for correlation analyses were assessed using the Shapiro–Wilk test. As the data did not follow normal distribution, associations were evaluated using Spearman’s rank correlation coefficient (*ρ*) (*n* = 8). Given the small number of analyzed samples, correlation analyses were considered exploratory, and statistical significance was set at *p* < 0.05. Correlations between total phenolic content and antioxidant activity (DPPH), and between the module of impedance, total phenolic content and total flavonoid content were assessed using Spearman’s rank correlation coefficient.

## 5. Conclusions

This study identified differences in the physicochemical and biological characteristics of propolis samples collected from four apiaries at two contrasting collection periods in the Zona da Mata region of Minas Gerais. Summer-collected samples generally exhibited higher dry extract and total phenolic content, lower wax levels, and stronger antioxidant responses in several complementary assays, whereas total flavonoid content and antibacterial activity against *S. aureus* remained comparatively stable. Qualitative UHPLC-ESI-MS/MS profiling revealed substantial chemical similarity among the samples and detected characteristic constituents of Brazilian green propolis, consistent with a possible contribution from *Baccharis dracunculifolia*. However, botanical origin cannot be conclusively established from chemical profiling alone. Electrical impedance measurements provided complementary information on physicochemical differences among the extracts and were negatively correlated with total phenolic and flavonoid content. Notably, D1 and D2 exhibited pronounced and selective cytotoxicity toward MDA-MB-231 cells, although their responses differed between the MTT and SRB assays. D2 also reduced colony-forming capacity and cell migration, supporting further investigation of its activity against triple-negative breast cancer cells. Overall, the differences observed between the two collection periods provide exploratory evidence of temporal variation in regional propolis. Nevertheless, broader longitudinal sampling is required to establish seasonal effects. Targeted quantitative chemical analyses will also be important to investigate the constituents associated with the biological activities observed.

## Figures and Tables

**Table 1:** Annotated compounds in propolis samples collected in the Zona da Mata region of Minas Gerais, Brazil, during summer and winter.

ID	Retention Time (min)	Molecular Formula	Annotated Compound	[M-H]^−^ (*m*/*z*)	MS/MS	Error (ppm)	Zona da Mata Region and Season
1	4.46	C_16_H_18_O_9_	Chlorogenic acid	353.0876	191.0549 179.0343 173.0439 135.0450	0.96	A1, A2, B1, B2, C1, C2, D1, D2
2	4.81	C_16_H_18_O_8_	Coumaroylquinic acid	337.0930	191.0549 179.0343 135.0450	1.95	A1, B1, B2
3	5.11	C_17_H_20_O_9_	*O*-Feruloylquinic acid	367.1040	191.0549 173.0439	2.96	A1, A2, B1, B2, C1, C2, D1, D2
4	5.24	C_9_H_8_O_3_	*p*-Coumaric acid	163.0397	119.0494	1.10	A1, A2, B1, B2, C2, D1, D2
5	5.79	C_25_H_24_O_12_	Dicaffeoylquinic acid	515.1183	353.0876 191.0549 179.0343 173.0439	−1.26	A1, A2, B1, B2, C1, C2, D1, D2
6	6.20	C_15_H_12_O_6_	Aromadendrin	287.0559	259.0610 125.0249	1.18	A1, A2, B1, B2, C1, C2, D1, D2
7	6.81	C_34_H_30_O_15_	Tricaffeoylquinic acid	677.1535	515.1183 353.0876 179.0343 173.0439	4.20	A1, A2, B1, B2, C1, C2, D2
8	7.03	C_16_H_12_O_7_	Rhamnetin	315.0501	300.0264 271.0226 165.9904	−1.20	A1, A2, B1, B2, C1, C2, D1, D2
9	7.50	C_15_H_10_O_6_	Kaempferol	285.0412	255.0281 179.0343 161.0232 151.0031	4.52	A1, A2, B1, B2, C1, C2, D1, D2
10	7.61	C_16_H_14_O_6_	Dihydrokaempferide	301.0731	283.0612 227.0696 152.0108 151.0031 125.0249	6.27	A1, A2, B1, B2, C1, C2, D1, D2
11	8.22	C_14_H_16_O_4_	Dihydroxyprenylcinnamic acid	247.0959	203.1071 148.0523	−4.57	A1, A2, B1, B2, C2, D1
12	8.45	C_14_H_16_O_3_	Drupanin	231.1030	187.1120 132.0577	3.80	A1, A2, B1, B2, C1, C2, D1, D2
13	8.63	C_19_H_24_O_4_	Capillartemisin A/B	315.1628	285.1483 271.1708 253.1587 198.1037 186.1054	1.60	A1, A2, B1, B2, D1, D2
14	8.95	C_15_H_12_O_4_	Pinocembrin	255.0674	213.0561 151.0031	6.54	A1, A2, B1, B2, C1, C2, D1, D2
15	9.04	C_16_H_12_O_6_	Kaempferide	299.0558	284.0331 164.0107 151.0031	0.80	A1, A2, B1, B2, C2, D1, D2
16	9.20	C_17_H_14_O_7_	Quercetin dimethyl ether	329.0658	314.0446 299.0558 271.0259 179.0343	−1.0	A1, A2, B1, B2, C1, C2, D1, D2
17	10.54	C_19_H_24_O_3_	Artepillin C	299.1655	255.1752	2.60	A1, A2, B1, B2, C1, C2, D1, D2

**ID:** compound number. **1:** Samples collected in the summer. **2:** Samples collected in the winter. **A:** Rancho Alegre Apiary. **B:** Belmiro Braga Apiary. **C:** Valadares Apiary. **D:** Salvaterra Apiary.

**Table 2:** Values of MIC for hydroethanolic extracts of propolis against *Staphylococcus aureus* (ATCC 6538).

Propolis Sample	Summer (µg/mL)	Winter (µg/mL)
**A**	500	500
**B**	500	>1000
**C**	500	500
**D**	250	250

Chloramphenicol (2–500 µg/mL) was used as the positive control. All propolis extracts exhibited MBC values > 1000 µg/mL, with no apparent differences between the two collection periods. **A:** Rancho Alegre Apiary. **B:** Belmiro Braga Apiary. **C:** Valadares Apiary. **D:** Salvaterra Apiary.

**Table 3:** 50% inhibitory concentration (IC_50_) values and selectivity indices (SIs) of propolis extracts on tumor and non-tumor cell lines after 48 h of treatment, determined by the MTT assay.

Propolis Extracts	Non-Tumor Cell Lines		Tumor Cell Lines														
	MCF10A (1)	PNT2 (2)	MCF7	SI (1)	SI (2)	MDA-MB-231	SI (1)	SI (2)	A-375	SI (1)	SI (2)	HepG2	SI (1)	SI (2)	U-87	SI (1)	SI (2)
**A1**	29.72	214.9	369.0	0.08	0.58	76.74	0.39	2.80	280.2	0.11	0.77	147.5	0.20	1.46	411.4	0.07	0.52
**A2**	23.28	125.2	117.3	0.20	1.07	15.34	1.52	8.16	91.04	0.26	1.38	98.01	0.24	1.28	138.8	0.17	0.90
**B1**	181.9	281.0	317.5	0.57	0.89	115.8	1.57	2.43	518.7	0.35	0.54	156.1	1.17	1.80	437.4	0.42	0.64
**B2**	29.44	248.1	349.2	0.08	0.71	66.36	0.44	3.74	279.5	0.11	0.89	117.3	0.25	2.12	369.6	0.08	0.67
**C1**	16.10	150.5	170.5	0.09	0.88	36.05	0.45	4.17	136.6	0.12	1.10	60.55	0.27	2.49	146.7	0.11	1.03
**C2**	31.98	101.0	132.8	0.24	0.76	37.09	0.86	2.72	136.9	0.23	0.74	57.76	0.55	1.75	146.9	0.22	0.69
**D1**	31.46	247.2	149.5	0.21	1.65	4.00	7.86	61.72	203.1	0.15	1.22	101.9	0.31	2.43	265.7	0.12	0.93
**D2**	11.31	137.2	74.25	0.15	1.85	36.55	0.31	3.75	107.0	0.11	1.28	43.54	0.26	3.15	117.8	0.10	1.16
**DOX**	0.72	1.59	0.29	2.48	1.85	2.67	0.26	0.59	0.18	4	8.8	0.11	6.54	14.45	1.64	0.44	0.97

**1**—Samples collected in the summer. **2**—Samples collected in the winter. **A:** Rancho Alegre Apiary. **B:** Belmiro Braga Apiary. **C:** Valadares Apiary. **D:** Salvaterra Apiary.

**Table 4:** 50% inhibitory concentration (IC_50_) values and selectivity indices (SIs) of propolis extracts on tumor and non-tumor cell lines after 48 h of treatment, as determined by the sulforhodamine B (SRB) assay.

Propolis Extracts	Non-Tumor Cell Lines		Tumor Cell Lines														
	MCF10A (1)	PNT2 (2)	MCF7	SI (1)	SI (2)	MDA-MB-231	SI (1)	SI (2)	A-375	SI (1)	SI (2)	HepG2	SI (1)	SI (2)	U-87	SI (1)	SI (2)
**A1**	525.4	370.9	51.23	10.25	7.24	23.17	22.67	16.01	196.7	2.67	1.89	165.1	3.18	2.25	209.1	2.51	1.77
**A2**	211.4	144.4	17.29	12.23	8.35	25.82	8.19	5.59	155.8	1.36	0.93	102.0	2.07	1.42	137.2	1.54	1.05
**B1**	>1000	187.7	33.12	>30.2	5.67	10.79	>92.7	17.40	194.7	>5.14	0.96	131.2	>7.62	1.43	283.8	>3.52	0.66
**B2**	>1000	174.1	5.286	>189	32.94	26.2	>38.2	6.65	259.0	>3.86	0.67	8.5	>117	20.5	235.2	>4.25	0.74
**C1**	135.7	93.52	15.6	8.70	5.99	35.19	3.86	2.66	138.3	0.98	0.68	92.79	1.46	1.01	131.9	1.03	0.71
**C2**	165.1	133.4	11.2	14.74	11.91	11.33	14.6	11.77	165.6	0.996	0.81	112.4	1.47	1.19	204.4	0.81	0.65
**D1**	186	87.19	16.16	11.51	5.40	8.584	21.67	10.16	87.5	2.13	1.00	54.93	3.39	1.59	97.66	1.9	0.89
**D2**	442.7	240.0	8.687	50.96	27.63	2.517	175.9	95.35	165.6	2.67	1.45	116.6	3.8	2.06	265.2	1.67	0.90

**1**—Samples collected in the summer. **2**—Samples collected in the winter. **A:** Rancho Alegre Apiary. **B:** Belmiro Braga Apiary. **C:** Valadares Apiary. **D:** Salvaterra Apiary.

## Data Availability

The original contributions presented in this study are included in the article. Further inquiries can be directed at the corresponding author.

## Supplementary Materials

The following supporting information can be downloaded at https://www.mdpi.com/article/10.3390/molecules31203611/s1. Figure S1. UHPLC-ESI-MS/MS analysis of sample A1 in negative ionization mode. Figure S2. UHPLC-ESI-MS/MS analysis of sample A2 in negative ionization mode. Figure S3. UHPLC-ESI-MS/MS analysis of sample B1 in negative ionization mode. Figure S4. UHPLC-ESI-MS/MS analysis of sample B2 in negative ionization mode. Figure S5. UHPLC-ESI-MS/MS analysis of sample C1 in negative ionization mode. Figure S6. UHPLC-ESI-MS/MS analysis of sample C2 in negative ionization mode. Figure S7. UHPLC-ESI-MS/MS analysis of sample D1 in negative ionization mode. Figure S8. UHPLC-ESI-MS/MS analysis of sample D2 in negative ionization mode Figure S9. MS/MS spectrum of compound with *m*/*z* 353.0876 [M-H]^−^. Figure S10. MS/MS spectrum of compound with *m*/*z* 337.0930 [M-H]^−^. Figure S11. MS/MS spectrum of compound with *m*/*z* 367.1040 [M-H]^−^. Figure S12. MS/MS spectrum of compound with *m*/*z* 163.0397 [M-H]^−^. Figure S13. MS/MS spectrum of compound with *m*/*z* 515.1183 [M-H]^−^. Figure S14. MS/MS spectrum of compound with *m*/*z* 287.0559 [M-H]^−^. Figure S15. MS/MS spectrum of compound with *m*/*z* 677.1535 [M-H]^−^. Figure S16. MS/MS spectrum of compound with *m*/*z* 285.0412 [M-H]^−^. Figure S17. MS/MS spectrum of compound with *m*/*z* 315.0501 [M-H]^−^. Figure S18. MS/MS spectrum of compound with *m*/*z* 301.0731 [M-H]^−^. Figure S19. MS/MS spectrum of compound with *m*/*z* 247.0959 [M-H]^−^. Figure S20. MS/MS spectrum of compound with *m*/*z* 231.1030 [M-H]^−^. Figure S21. MS/MS spectrum of compound with *m*/*z* 315.1628 [M-H]^−^. Figure S22. MS/MS spectrum of compound with *m*/*z* 255.0674 [M-H]^−^. Figure S23. MS/MS spectrum of compound with *m*/*z* 299.0558 [M-H]^−^. Figure S24. MS/MS spectrum of compound with *m*/*z* 329.0658 [M-H]^−^. Figure S25. MS/MS spectrum of compound with *m*/*z* 299.1655 [M-H]^−^. Table S1. Physicochemical characteristics of the propolis samples. Table S2. Electrical impedance of propolis samples at 20 kHz frequency.

## Abbreviations

The following abbreviations are used in this manuscript:

APIJUR	Association of Beekeepers of Juiz de Fora and Region
DPPH	(2,2-diphenyl-1-picrylhydrazyl) radical
NO	Nitric Oxide
ORAC	Oxygen Radical Absorbance Capacity
SNP	Sodium nitroprusside
NED	N-(naphthyl) ethylenediamine
