# Supplementary material for: Variation in the Chemical Composition and Biological Activities of Propolis Collected During Two Seasonal Periods in the Zona da Mata Region of Brazil
Source: Molecules. 2026 Oct 10;31(20):3611. doi: 10.3390/molecules31203611 (PMC13707419; doi:10.3390/molecules31203611)
Supplement: Supplementary file 1 [file molecules-31-03611-s001.zip › molecules-4567139-supplementary.pdf]

Supplementary Material

Seasonal Variation in the Chemical Composition and Biological Activities of Propolis from the Zona da Mata Region of Brazil

Laís A. Moreira1[1], João Paulo B. Sousa[2], Carla Monteiro Leal[3], Victor Campana Leite[4], Jessica Pereira Silva[4], Maria Luiza A. A. Soares[4], Gislaine Gonçalves Rocha[5], Paula Marynella Alves Pereira Lima[5], Fabíola Dutra Rocha[1], Wesley W. G. Nascimento[6], Nicolas C. C. Pinto[4], Humberto Moreira Húngaro[1], Thaise Gonçalves de Araújo[5], Ademar A. Da Silva Filho[1]*

1 Departamento de Ciencias Farmaceuticas, Faculdade de Farmacia, Universidade Federal de Juiz de Fora, R. Jose Lourenco Kelmer s/n, Campus Universitario, Juiz de Fora 36036-900, MG, Brazil; lais.moreira@estudante.ufjf.br (L.A.M.); fabiola.rocha@ufjf.br (F.D.R.); humbertomh@gmail.com (H.M.H.)

2 Departamento de Farmacia, Faculdade de Farmacia, Universidade de Brasilia, Asa Norte, Campus Darcy Ribeiro, Brasilia 70910-900, DF, Brazil; jpsousa595@gmail.com

3 Departamento de Produtos Naturais e Alimentos, Faculdade de Farmacia, Universidade Federal do Rio de Janeiro, Av. Carlos Chagas Filho, 373, Cidade Universitaria, Rio de Janeiro 21941-902, RJ, Brazil; farmaceuticacarlaleal@gmail.com

4 Laboratorio de Produtos Naturais Bioativos, Departamento de Bioquimica, Instituto de Ciencias Biologicas, Universidade Federal de Juiz de Fora, R. Jose Lourenco Kelmer s/n, Campus Universitario, Juiz de Fora 36036-900, MG, Brazil; victor.campana@ufjf.br (V.C.L.); jessicapereira.silva@estudante.ufjf.br (J.P.S.); marialuiza.aasoares@gmail.com (M.L.A.A.S.); nicolas.pinto@ufjf.br (N.d.C.C.P.)

5 Laboratorio de Genetica e Biotecnologia, Instituto de Biotecnologia, Universidade Federal de Uberlandia, R. Major Jeronimo, 566, Patos de Minas 38700-002, MG, Brazil; gislaineg.rocha08@gmail.com (G.G.R.); paulamapl0607@gmail.com (P.M.A.P.L.); thaisegaraujo@gmail.com (T.G.A.)

6 Departamento de Farmacia, Instituto de Ciencias da Vida, Universidade Federal de Juiz de Fora, R. Manoel Byrro, 241, Campus Governador Valadares, Governador Valadares 35032-620, MG, Brazil; wesley.nascimento@ufjf.br

* Correspondence: ademar.alves@ufjf.br; Tel.: +55-32-2102-3893; Fax: +55-32-2102-3801

List of content:

Figure S1. UHPLC-ESI-MS/MS analysis of sample A1 in negative ionization mode.

Figure S2. UHPLC-ESI-MS/MS analysis of sample A2 in negative ionization mode.

Figure S3. UHPLC-ESI-MS/MS analysis of sample B1 in negative ionization mode.

Figure S4. UHPLC-ESI-MS/MS analysis of sample B2 in negative ionization mode.

Figure S5. UHPLC-ESI-MS/MS analysis of sample C1 in negative ionization mode.

Figure S6. UHPLC-ESI-MS/MS analysis of sample C2 in negative ionization mode.

Figure S7. UHPLC-ESI-MS/MS analysis of sample D1 in negative ionization mode.

Figure S8. UHPLC-ESI-MS/MS analysis of sample D2 in negative ionization mode

Figure S9. MS/MS spectrum of compound with *m*/*z* 353.0876 $[M-H]^-$.

Figure S10. MS/MS spectrum of compound with *m*/*z* 337.0930 $[M-H]^-$.

Figure S11. MS/MS spectrum of compound with *m*/*z* 367.1040 $[M-H]^-$.

Figure S12. MS/MS spectrum of compound with *m*/*z* 163.0397 $[M-H]^-$.

Figure S13. MS/MS spectrum of compound with *m*/*z* 515.1183 $[M-H]^-$.

Figure S14. MS/MS spectrum of compound with *m*/*z* 287.0559 $[M-H]^-$.

Figure S15. MS/MS spectrum of compound with *m*/*z* 677.1535 $[M-H]^-$.

Figure S16. MS/MS spectrum of compound with *m*/*z* 285.0412 $[M-H]^-$.

Figure S17. MS/MS spectrum of compound with *m*/*z* 315.0501 $[M-H]^-$.

Figure S18. MS/MS spectrum of compound with *m*/*z* 301.0731 $[M-H]^-$.

Figure S19. MS/MS spectrum of compound with *m/z* 247.0959 $[M-H]^-$.
Figure S20. MS/MS spectrum of compound with *m/z* 231.1030 $[M-H]^-$.
Figure S21. MS/MS spectrum of compound with *m/z* 315.1628 $[M-H]^-$.
Figure S22. MS/MS spectrum of compound with *m/z* 255.0674 $[M-H]^-$.
Figure S23. MS/MS spectrum of compound with *m/z* 299.0558 $[M-H]^-$.
Figure S24. MS/MS spectrum of compound with *m/z* 329.0658 $[M-H]^-$.
Figure S25. MS/MS spectrum of compound with *m/z* 299.1655 $[M-H]^-$.
Table S1. Physicochemical characteristics of the propolis samples.
Table S2. Electrical impedance of propolis samples at 20 kHz frequency

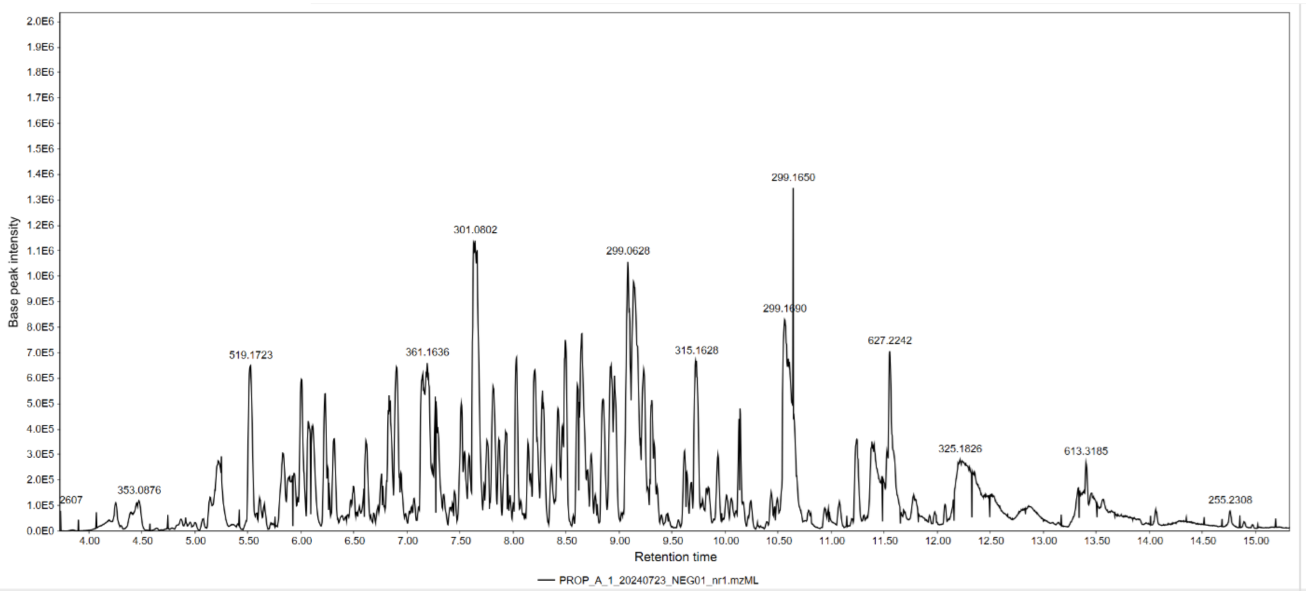

Figure S1. UHPLC-ESI-MS/MS analysis of sample A1 in negative ionization mode**.**

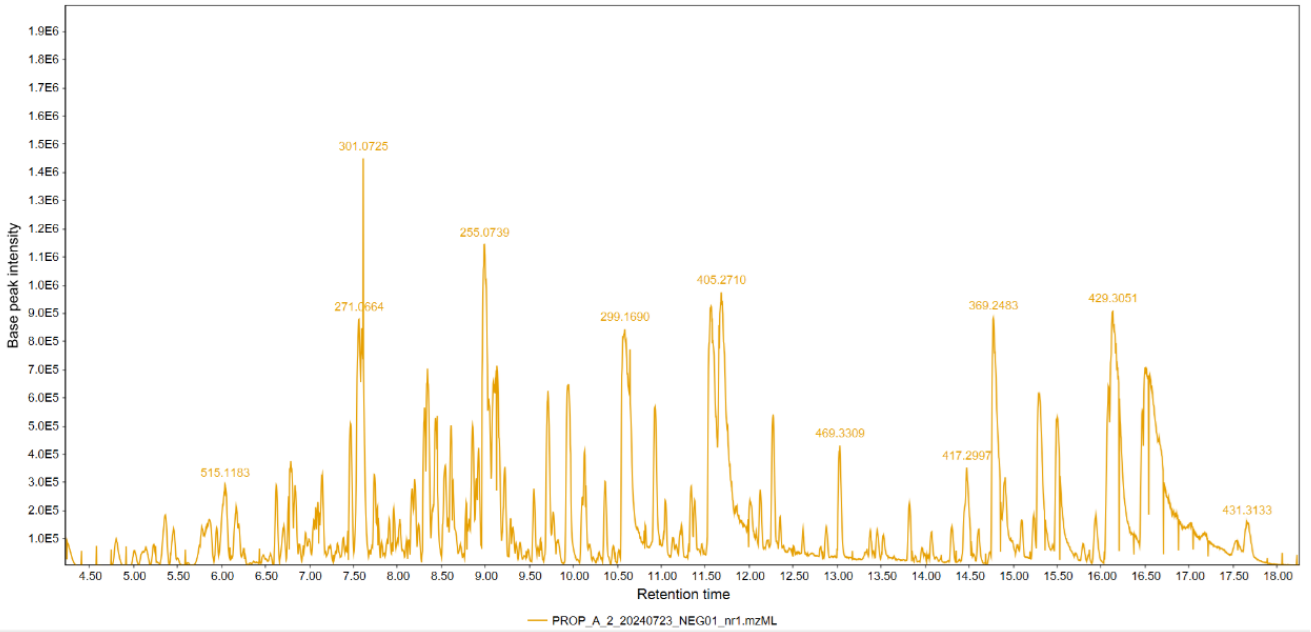

Figure S2. UHPLC-ESI-MS/MS analysis of sample A2 in negative ionization mode.

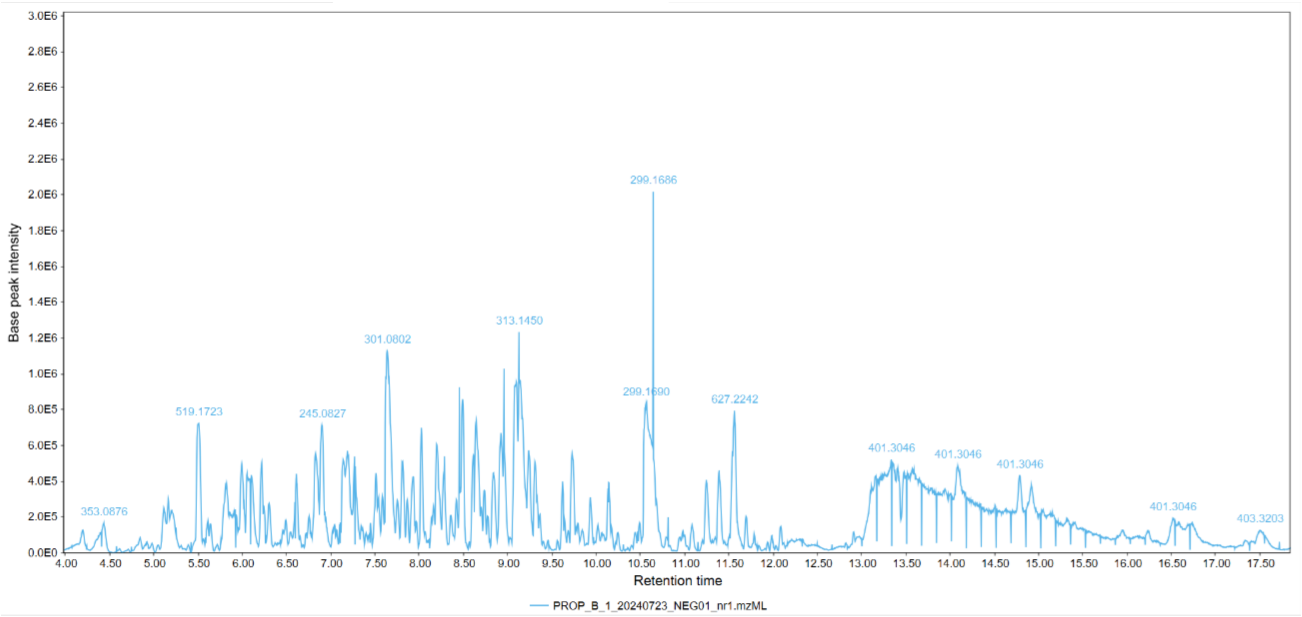

Figure S3. UHPLC-ESI-MS/MS analysis of sample B1 in negative ionization mode.

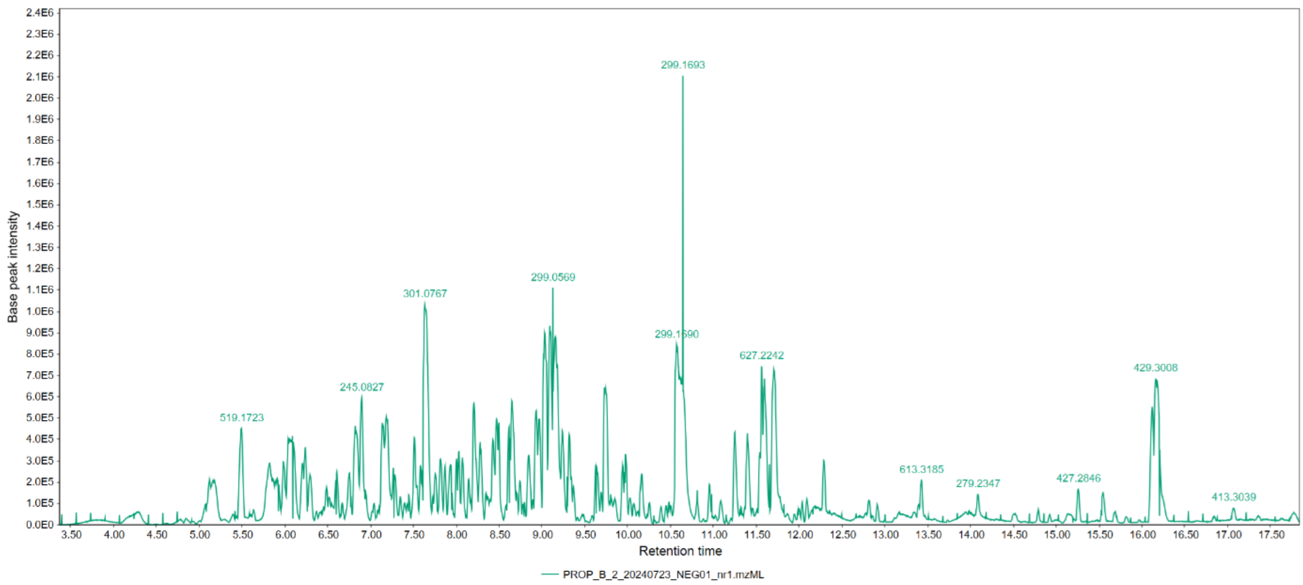

Figure S4. UHPLC-ESI-MS/MS analysis of sample B2 in negative ionization mode.

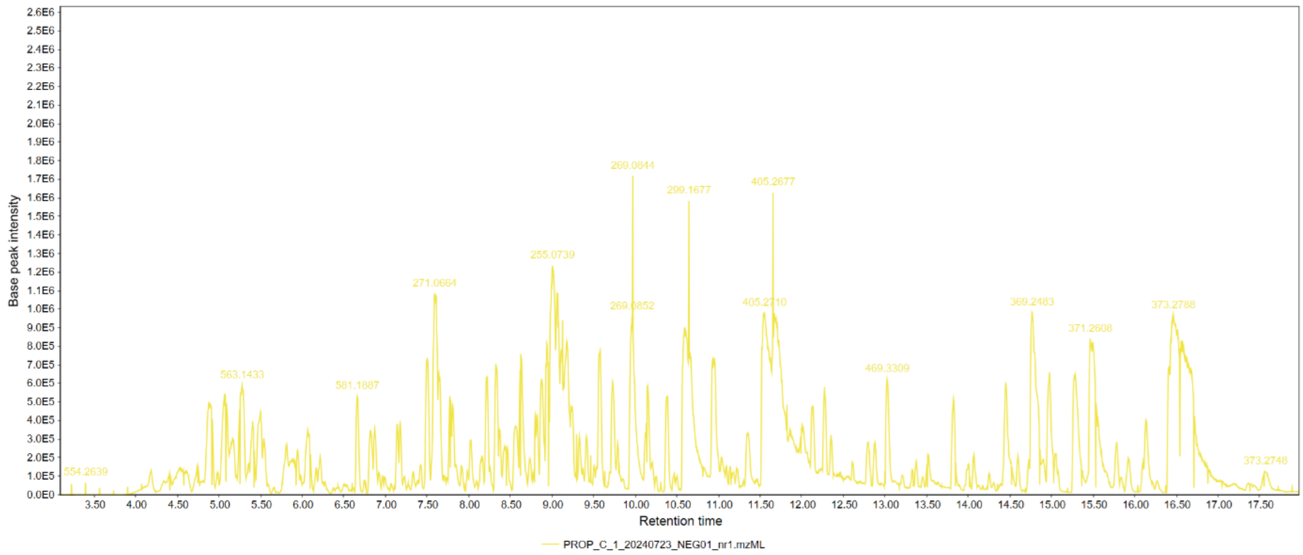

Figure S5. UHPLC-ESI-MS/MS analysis of sample C1 in negative ionization mode.

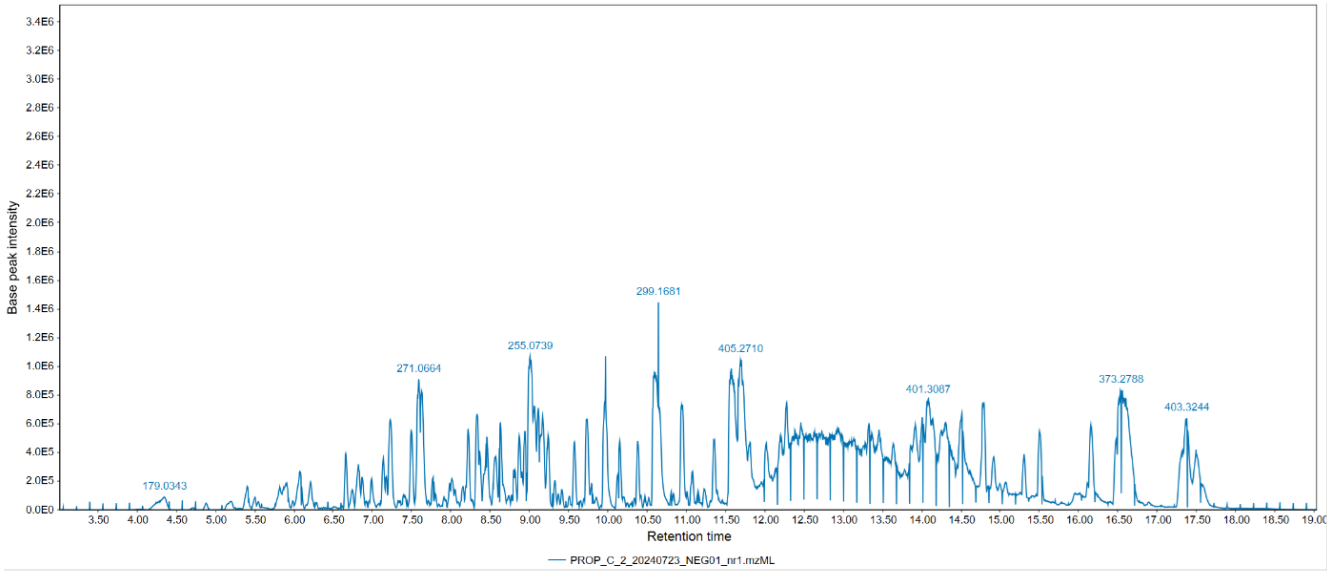

Figure S6. UHPLC-ESI-MS/MS analysis of sample C2 in negative ionization mode.

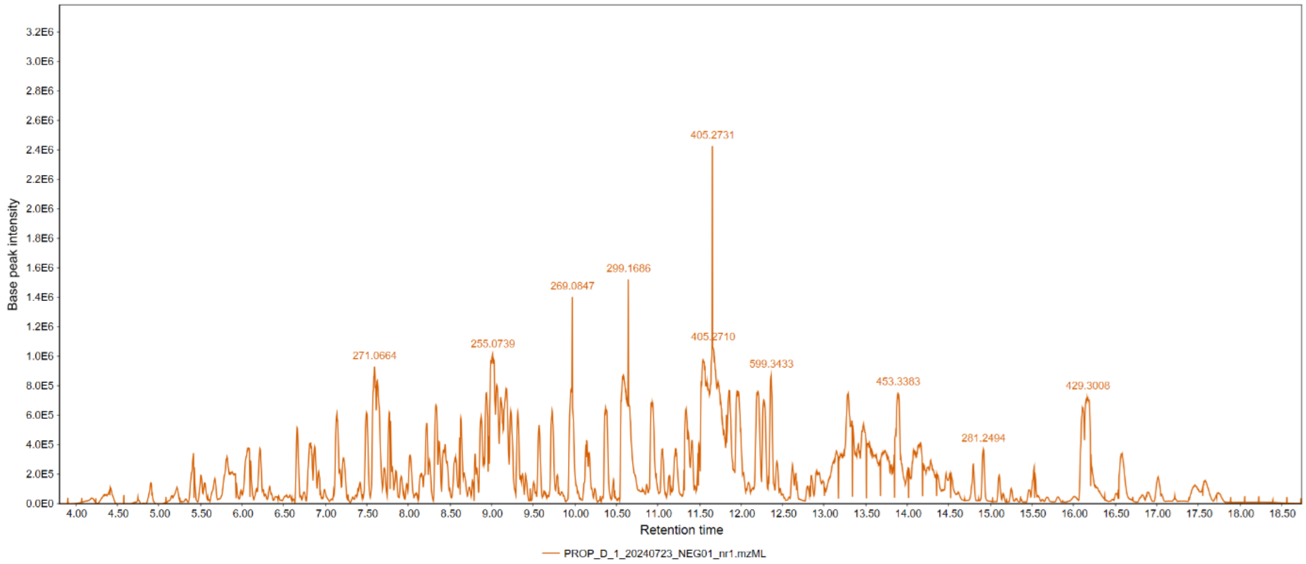

Figure S7. UHPLC-ESI-MS/MS analysis of sample D1 in negative ionization mode.

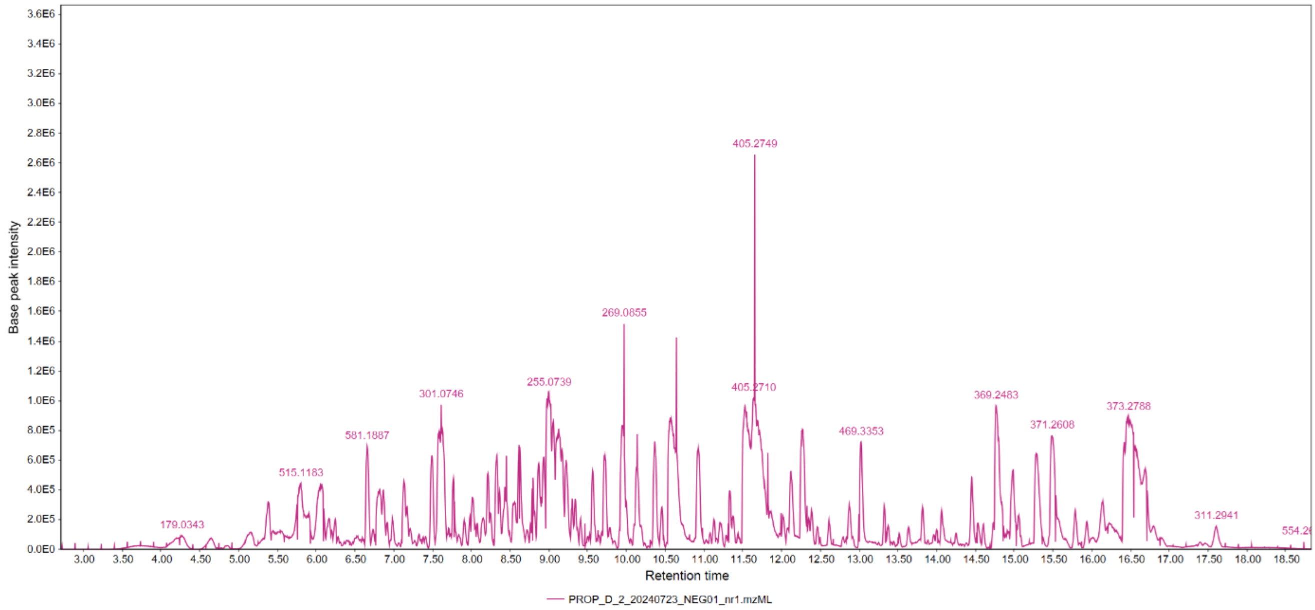

Figure S8. UHPLC-ESI-MS/MS analysis of sample D2 in negative ionization mode.

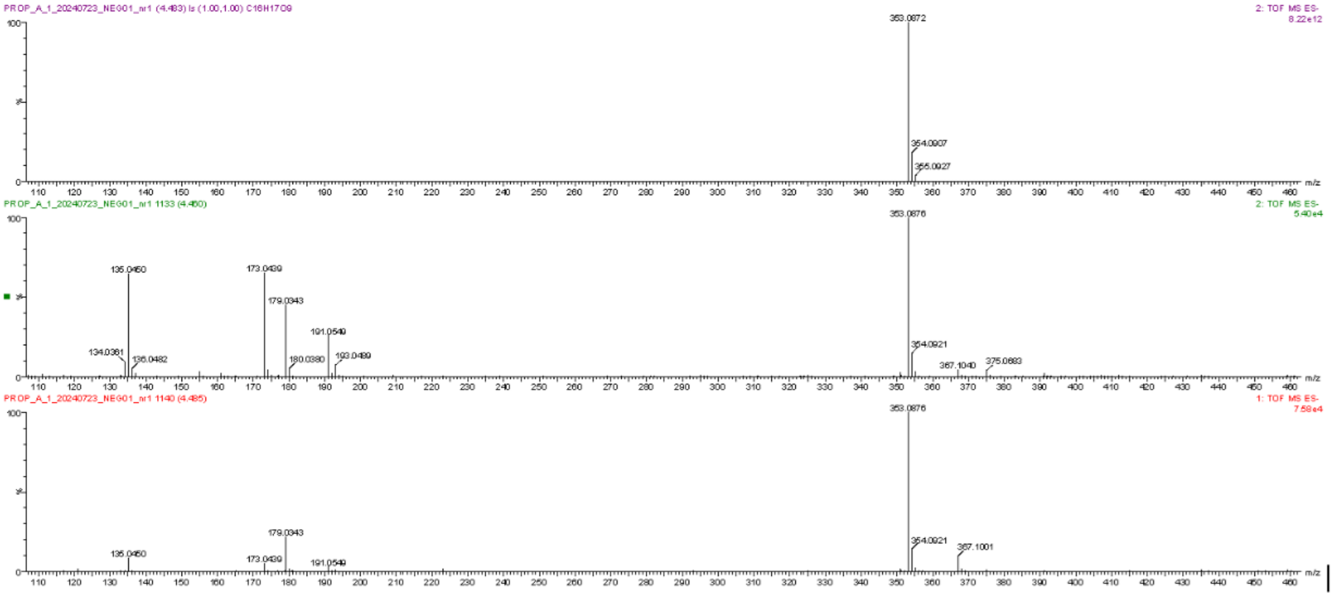

Figure S9. MS/MS spectrum of compound with *m*/*z* 353.0876 [M-H]$^-$.

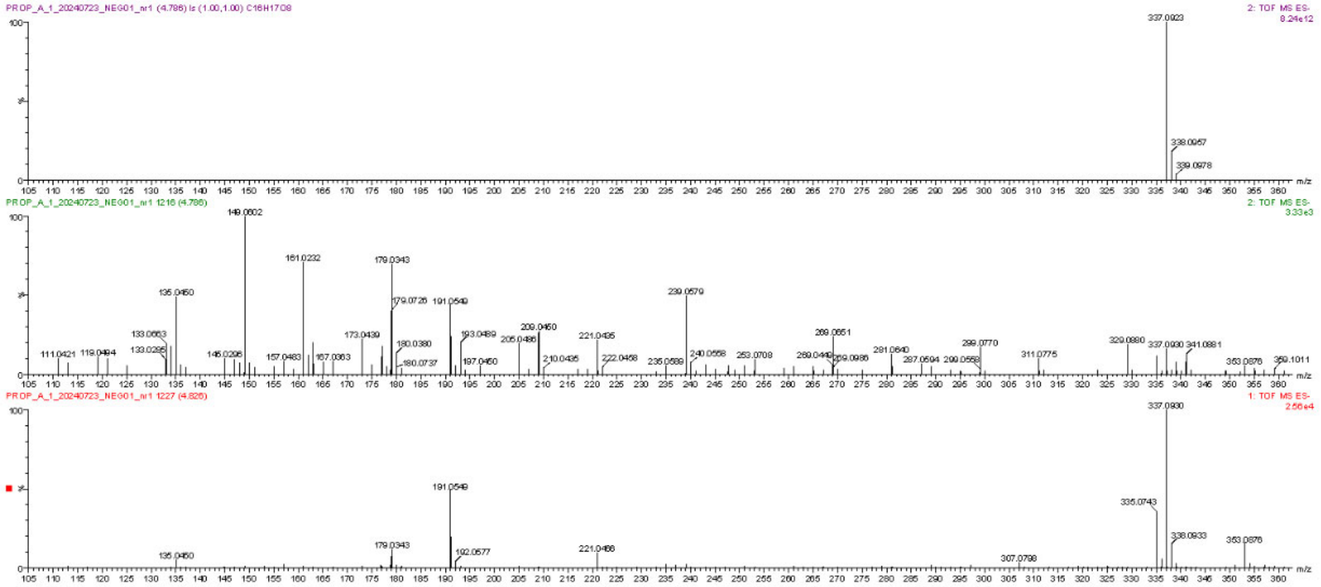

Figure S10. MS/MS spectrum of compound with *m*/*z* 337.0930 [M-H]$^-$.

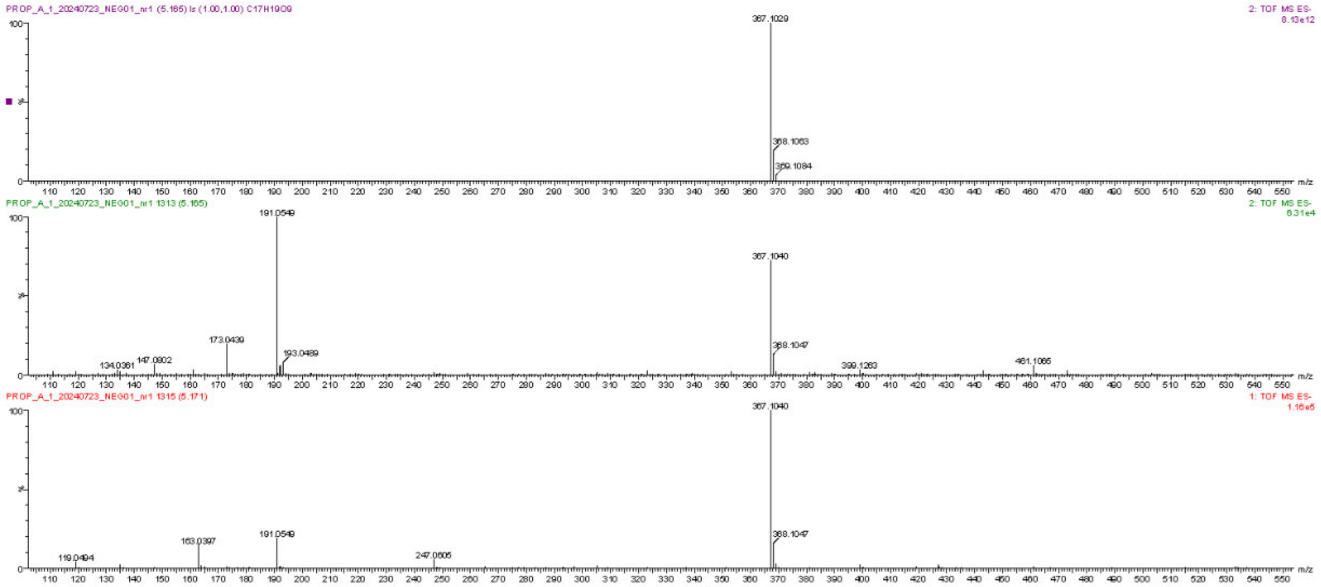

Figure S11. MS/MS spectrum of compound with *m*/*z* 367.1040 [M-H]⁻.

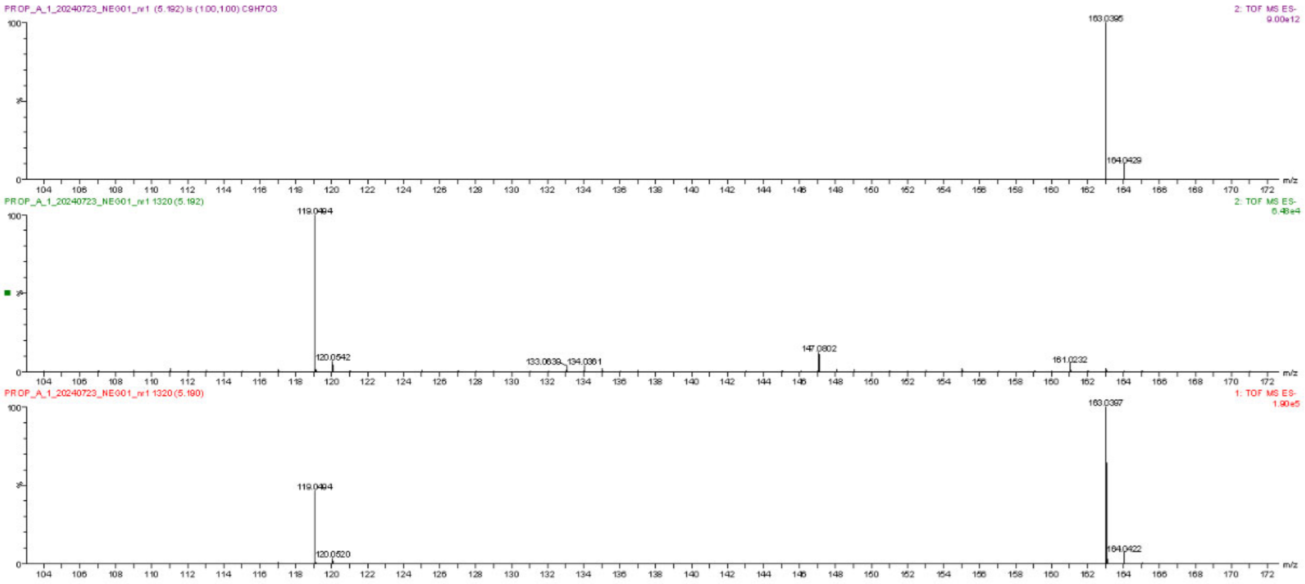

Figure S12. MS/MS spectrum of compound with *m*/*z* 163.0397 [M-H]⁻.

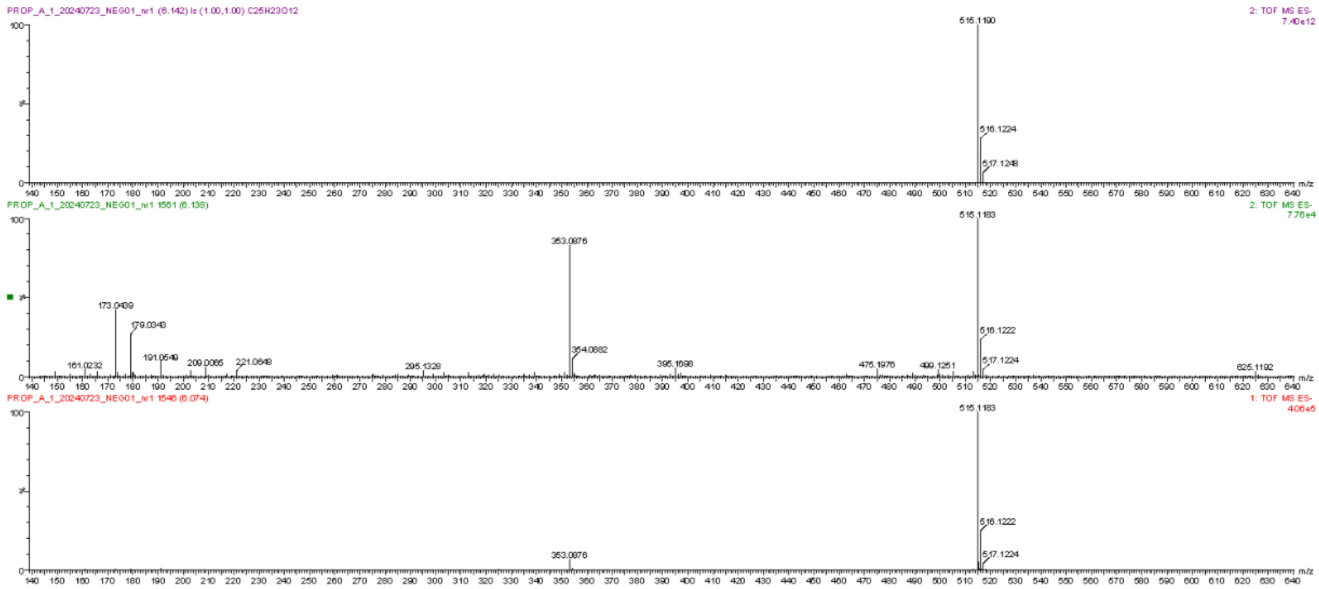

Figure S13. MS/MS spectrum of compound with *m*/*z* 515.1183 [M-H]-.

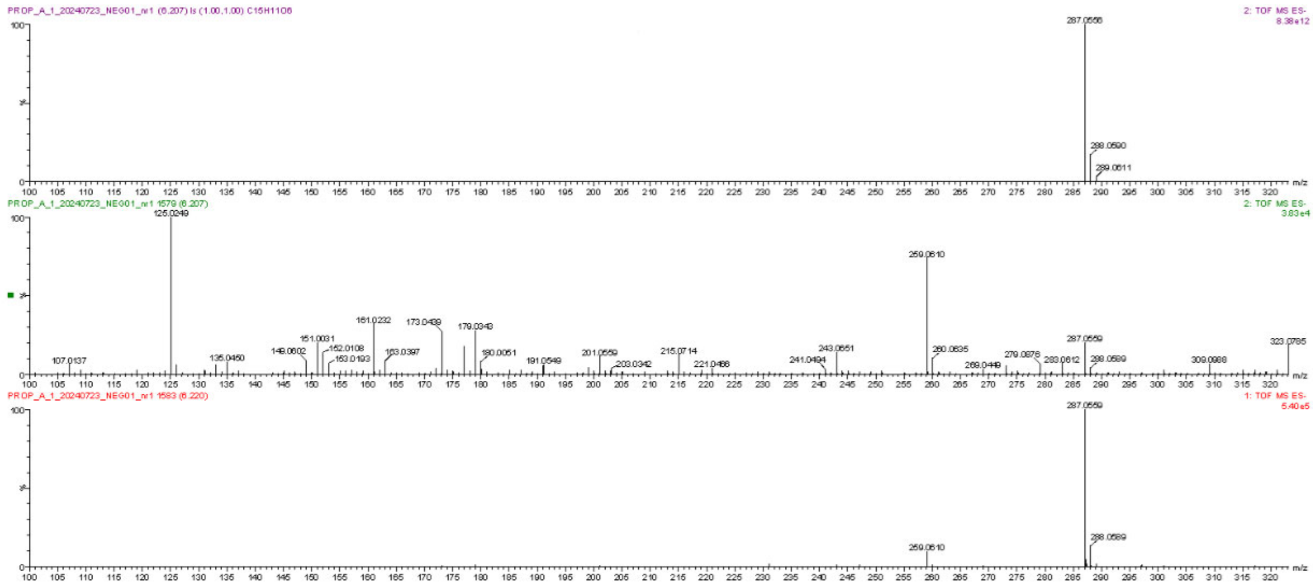

Figure S14. MS/MS spectrum of compound with *m*/*z* 287.0559 [M-H]-.

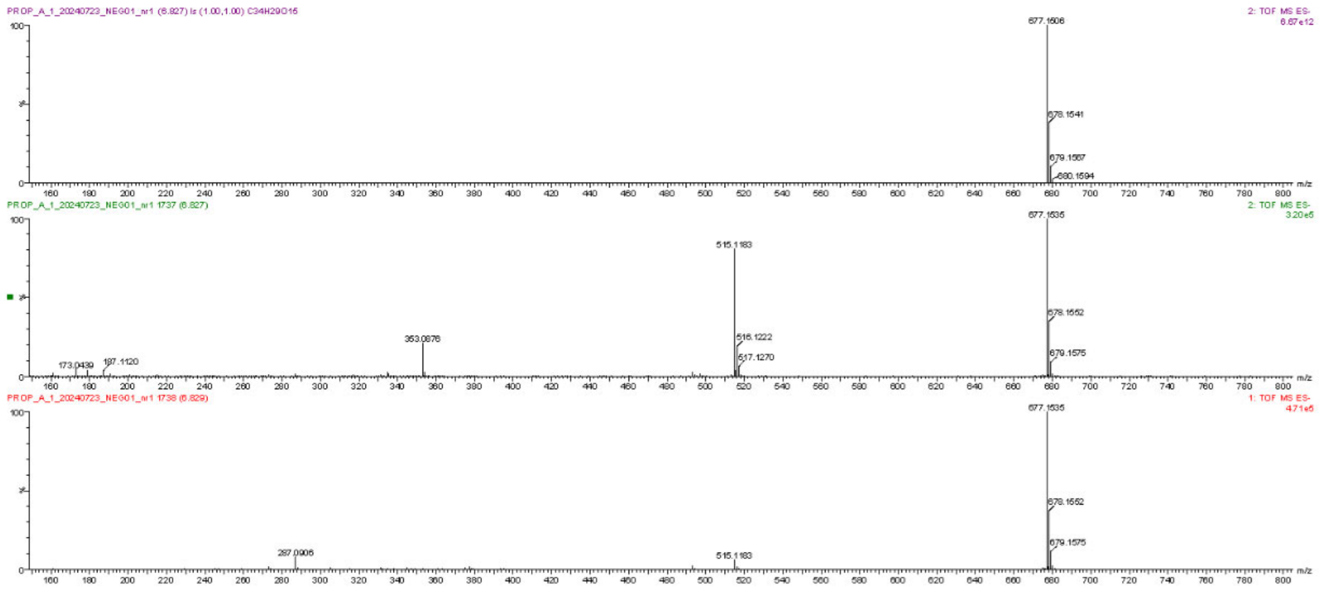

Figure S15. MS/MS spectrum of compound with *m/z* 677.1535 [M-H]⁻.

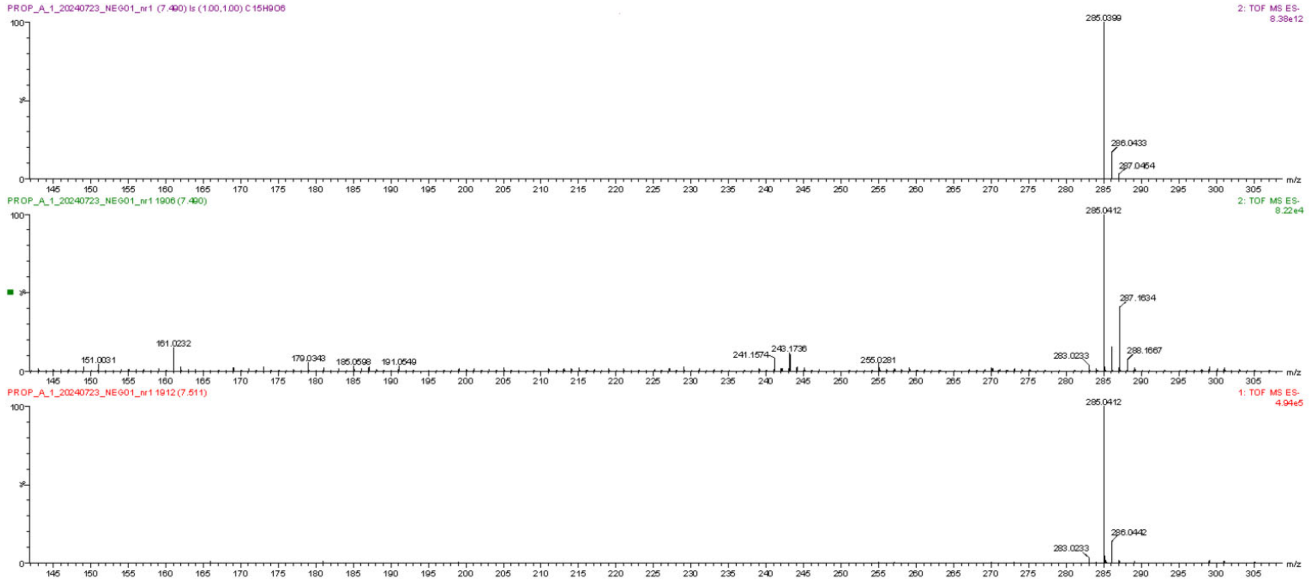

Figure S16. MS/MS spectrum of compound with *m/z* 285.0412 [M-H]⁻.

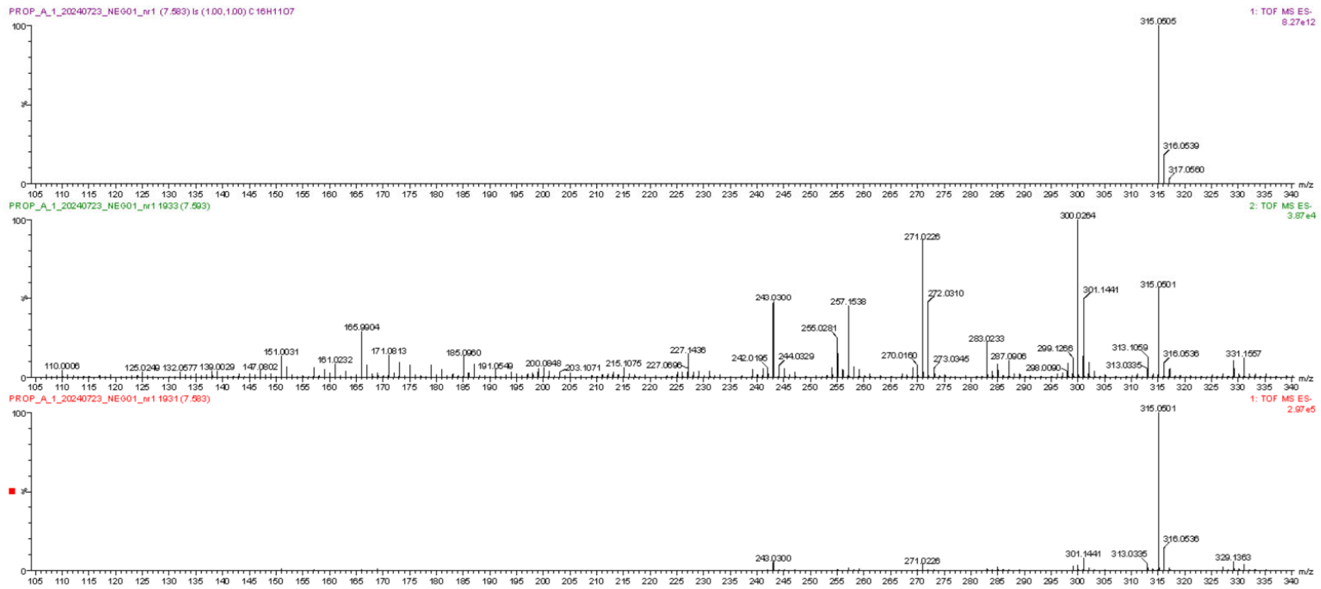

Figure S17. MS/MS spectrum of compound with *m*/*z* 315.0501 [M-H]$^-$.

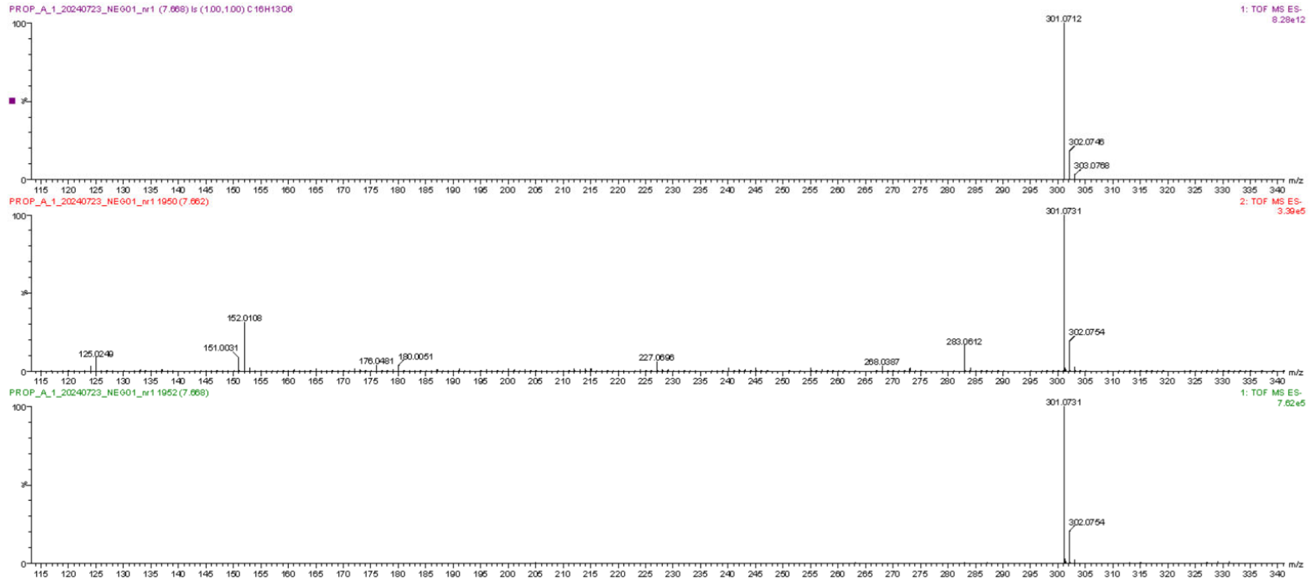

Figure S18. MS/MS spectrum of compound with *m*/*z* 301.0731 [M-H]$^-$.

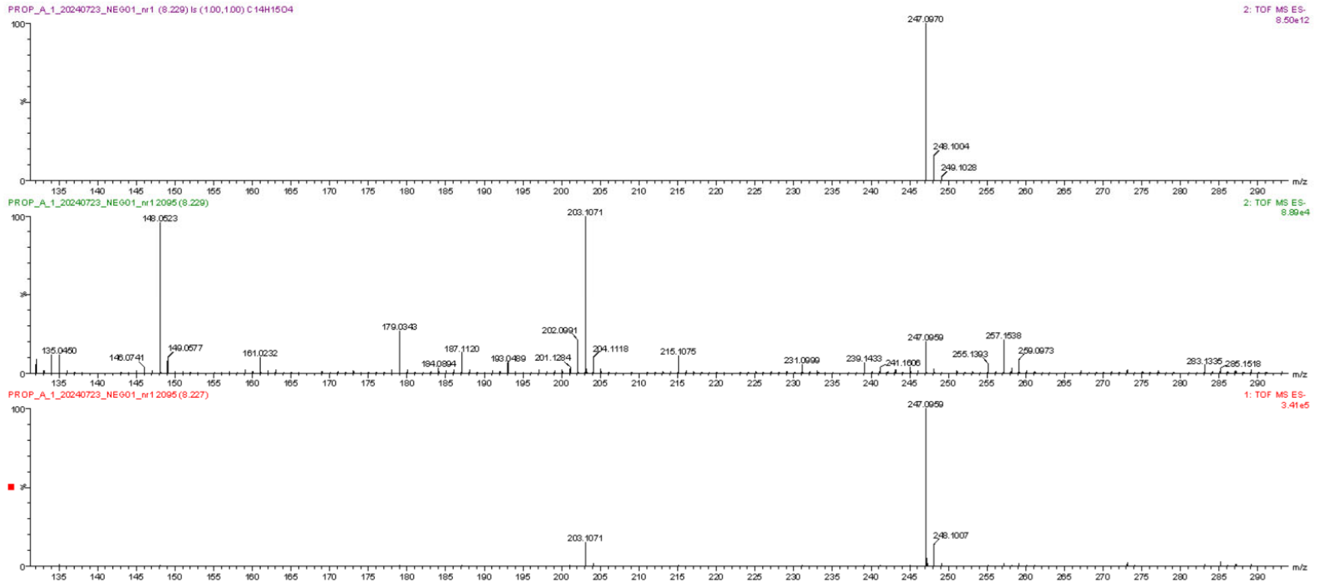

Figure S19. MS/MS spectrum of compound with *m/z* 247.0959 [M-H]-.

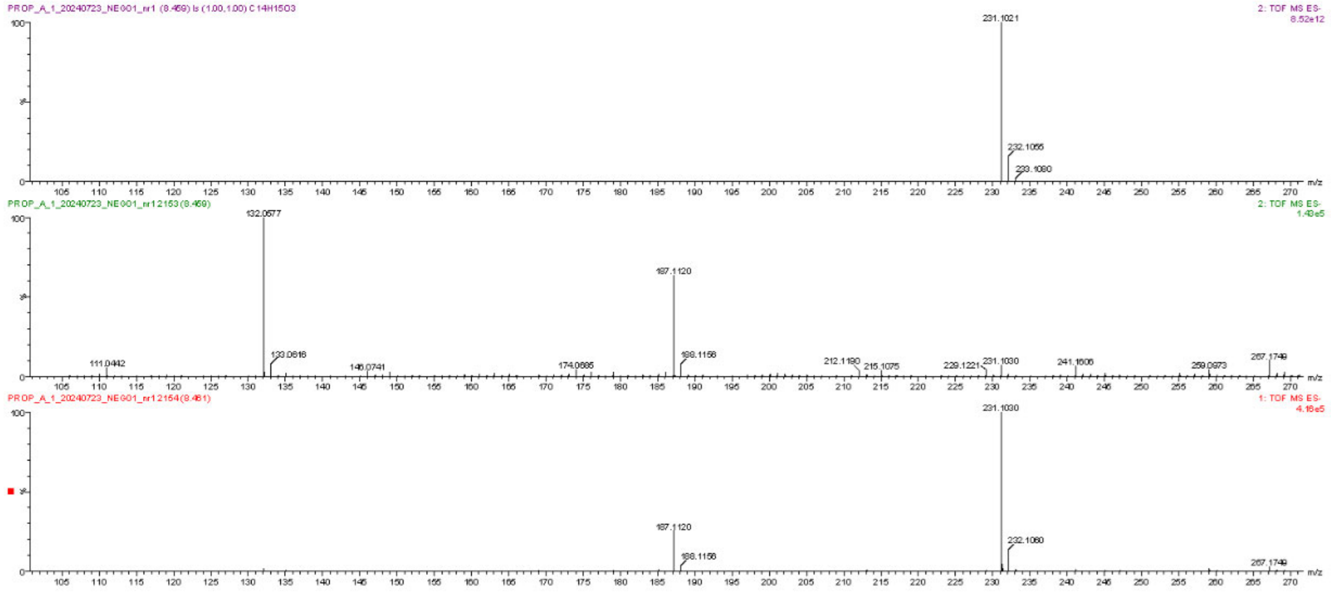

Figure S20. MS/MS spectrum of compound with *m/z* 231.1030 [M-H]-.

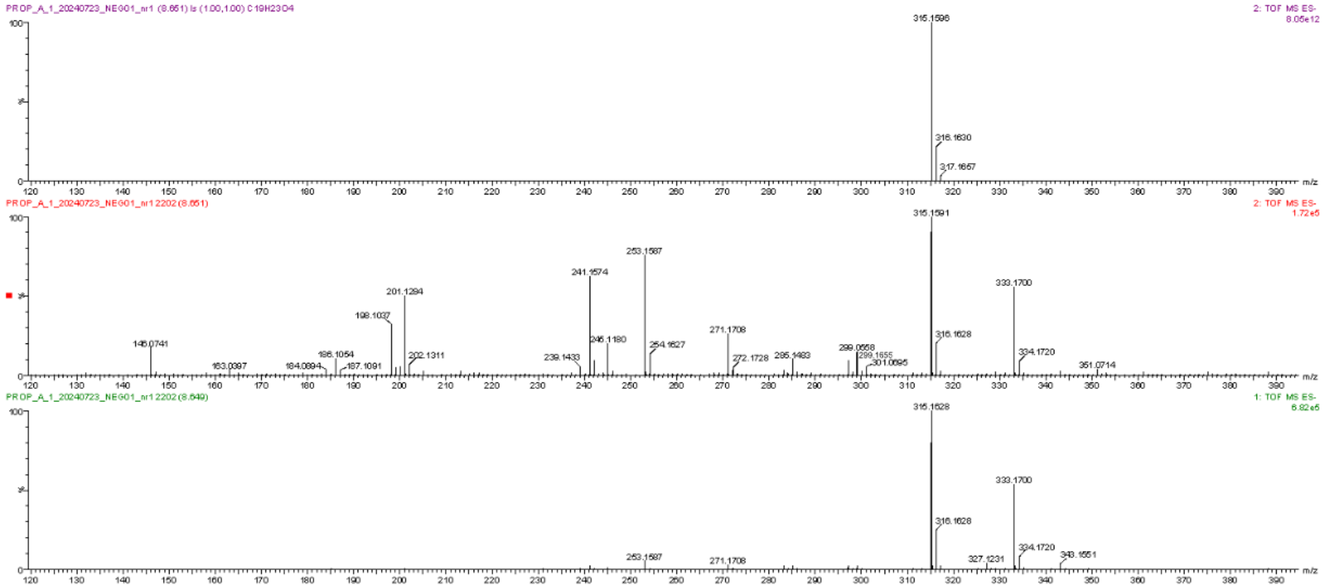

Figure S21. MS/MS spectrum of compound with *m/z* 315.1628 [M-H]$^-$.

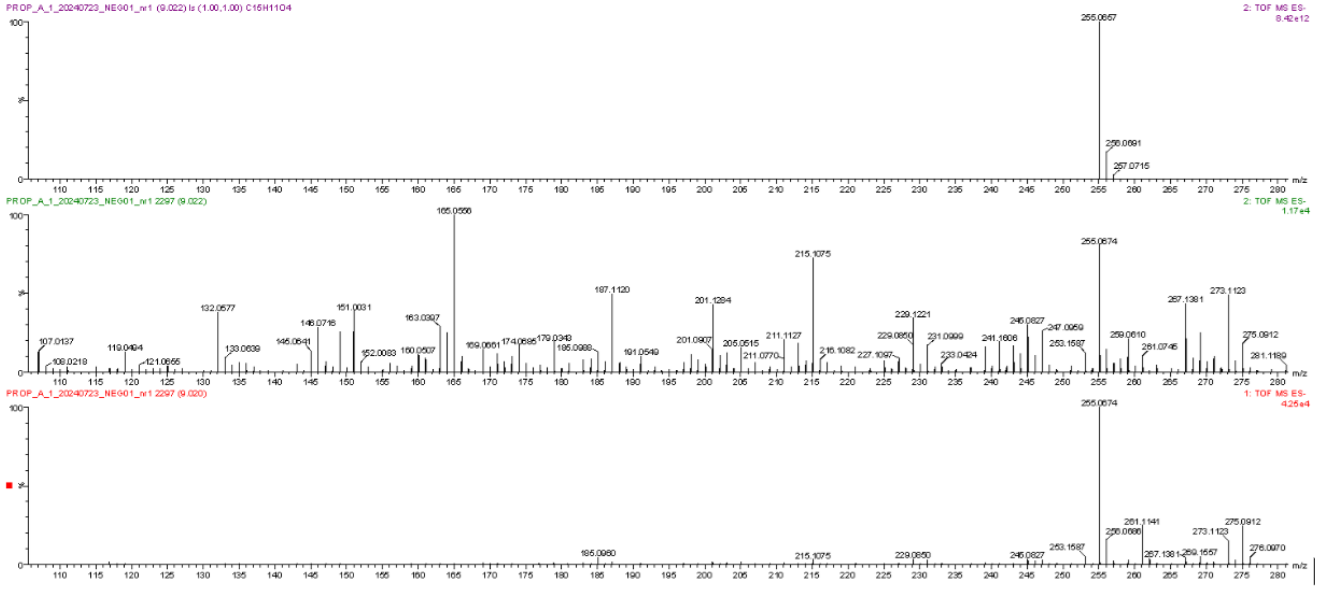

Figure S22. MS/MS spectrum of compound with *m/z* 255.0674 [M-H]$^-$.

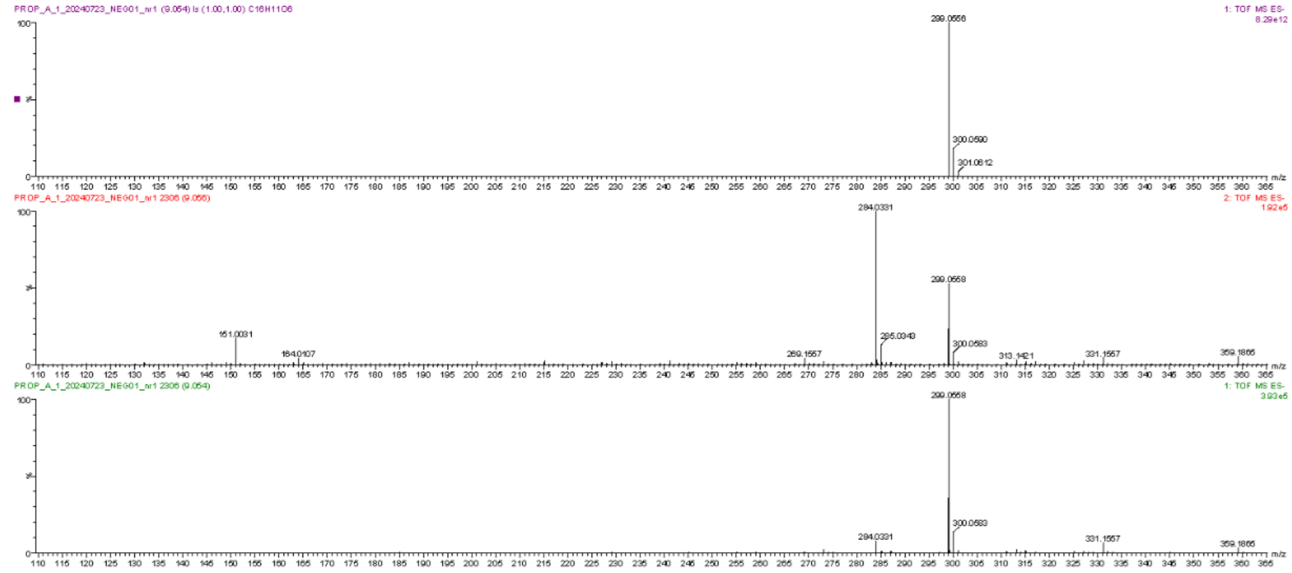

Figure S23. MS/MS spectrum of compound with *m*/*z* 299.0558 [M-H]⁻.

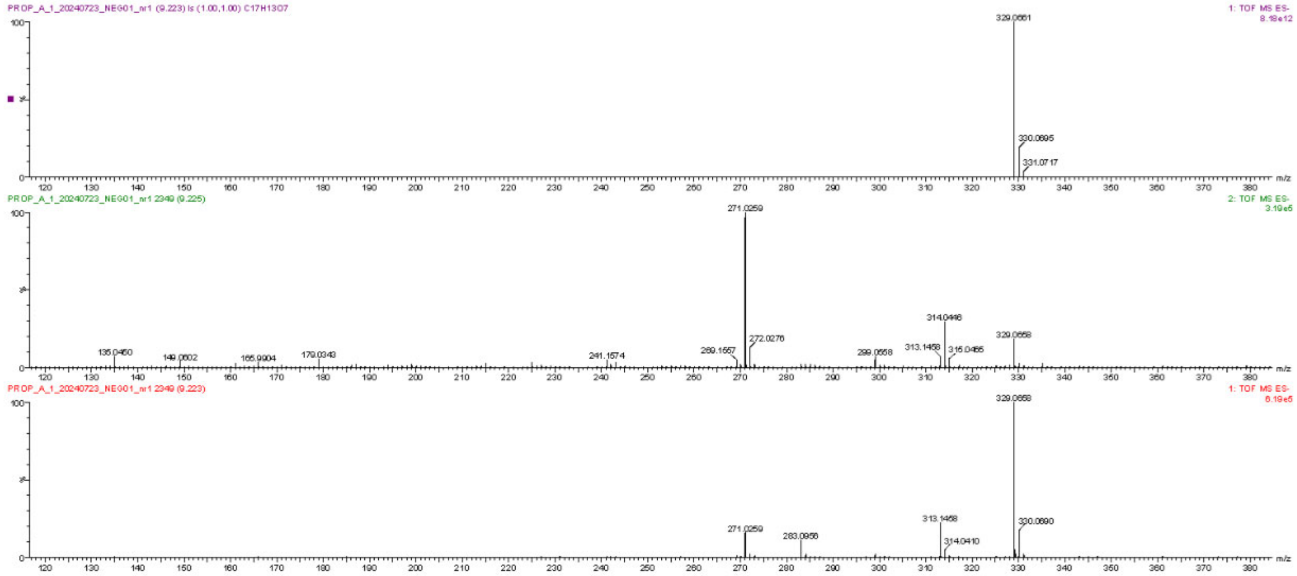

Figure S24. MS/MS spectrum of compound with *m*/*z* 329.0658 [M-H]⁻.

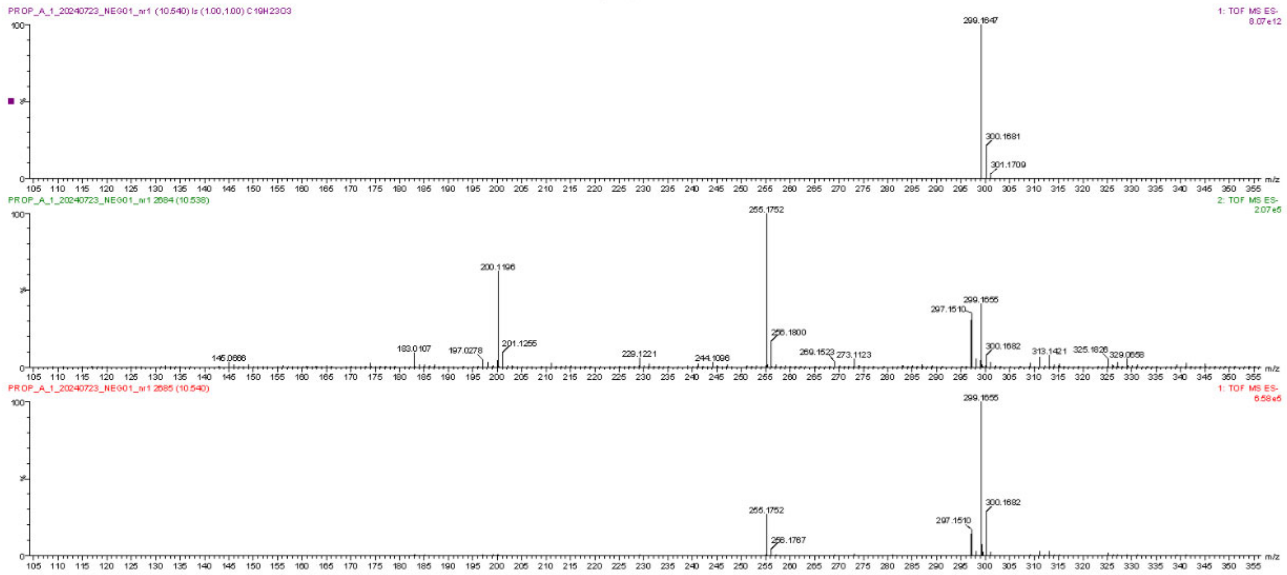

Figure S25. MS/MS spectrum of compound with m/z 299.1655 $[M-H]^-$.

Table S1. Physicochemical characteristics of the propolis samples.

Propolis Sample	Waxes (%)	Yeld (mg/g of raw propolis)
**A1**	0.61 ± 0.57[a]	642.7 ± 15[a]
**A2**	0.91 ± 2.02[a]	411.8 ± 23[b]
**B1**	1.34 ± 0.57[a]	730.2 ±25[c]
**B2**	6.46 ± 2.02[b]	634.7 ± 10[a]
**C1**	2.24 ± 0.57[c]	331.1 ± 35[d]
**C2**	2.48 ± 2.02[c]	263.3 ± 12[d]
**D1**	1.45 ± 0.57[a]	666.0 ± 10[a]
**D2**	2.99 ± 2.02[c]	587.4 ± 8.0[a]

*Results are presented as mean ± SEM of three independent experiments.

Table S2. Electrical impedance of propolis samples at 20 kHz frequency

Propolis Sample	Impedance (kΩ)
**A1**	11.286
**A2**	20.731
**B1**	14.366
**B2**	17.252
**C1**	14.907
**C2**	15.896
**D1**	13.974
**D2**	19.366
